# A Systematic Literature Review on Machine Learning and Deep Learning Approaches for Detecting DDoS Attacks in Software-Defined Networking

**DOI:** 10.3390/s23094441

**Published:** 2023-05-01

**Authors:** Abdullah Ahmed Bahashwan, Mohammed Anbar, Selvakumar Manickam, Taief Alaa Al-Amiedy, Mohammad Adnan Aladaileh, Iznan H. Hasbullah

**Affiliations:** 1 National Advanced IPv6 Centre (NAv6), Universiti Sains Malaysia, Gelugor 11800, Penang, Malaysia; 2 Cybersecurity Department, School of Information Technology, American University of Madaba (AUM), Amman 11821, Jordan

**Keywords:** systematic literature review (SLR), software-defined networking (SDN), machine learning (ML), deep learning (DL), distributed denial of service (DDoS), intrusion detection system (IDS)

## Abstract

Software-defined networking (SDN) is a revolutionary innovation in network technology with many desirable features, including flexibility and manageability. Despite those advantages, SDN is vulnerable to distributed denial of service (DDoS), which constitutes a significant threat due to its impact on the SDN network. Despite many security approaches to detect DDoS attacks, it remains an open research challenge. Therefore, this study presents a systematic literature review (SLR) to systematically investigate and critically analyze the existing DDoS attack approaches based on machine learning (ML), deep learning (DL), or hybrid approaches published between 2014 and 2022. We followed a predefined SLR protocol in two stages on eight online databases to comprehensively cover relevant studies. The two stages involve automatic and manual searching, resulting in 70 studies being identified as definitive primary studies. The trend indicates that the number of studies on SDN DDoS attacks has increased dramatically in the last few years. The analysis showed that the existing detection approaches primarily utilize ensemble, hybrid, and single ML-DL. Private synthetic datasets, followed by unrealistic datasets, are the most frequently used to evaluate those approaches. In addition, the review argues that the limited literature studies demand additional focus on resolving the remaining challenges and open issues stated in this SLR.

## 1. Introduction

In the last decade, the network infrastructure has seen a rapid expansion of network devices, which increases administrative complexity and creates obstacles to future Internet innovation. Moreover, the rigidity of the conventional network decreases elasticity and increases operational expenses. As a result, those challenges impede the momentum of emerging technologies, including cloud computing, IoT technologies, and big data, which progressively demand more bandwidth, flexibility, and manageability [1,2]. Under those circumstances, SDN [3] emerged and was touted as an innovative networking model capable of handling and addressing the growing demands of next-generation networks and emerging technologies.

Furthermore, the most critical and distinguishing feature of SDN architecture vs. traditional network architecture is decoupling the logical control plane from the data plane, where the centralized control plane controls several distributed network devices (i.e., routers and switches). This feature has many beneficial advantages, including having a global view of the entire network via a centralized controller, providing programmable and standard interfaces, enhancing the management of switches, efficient construction of virtual logical networks, and allowing centralized monitoring modules. The SDN’s elasticity, programmability, scalability, and manageability features enable it to make its way into enterprise networks, wireless networks, backbone networks, and data centers [4].

The SDN architecture contains three planes or layers: application, control, and data. The application plane hosts various business applications implemented by developers, such as traffic engineering and network-monitoring apps. The control plane, where the SDN controller resides, typically comprises single or multiple logically centralized controllers for managing the data plane using southbound application programming interfaces (APIs) and providing the application plane with network services through northbound APIs. Finally, the data plane mainly comprises network forwarding devices. The SDN relies on the OpenFlow protocol [5], which plays a vital role in providing a central control and holistic view of the entire network. It is also preferred and widely used for communication between SDN controllers and OpenFlow switches [6].

Historically, SDN and OpenFlow started purely as academic and scientific research endeavors but have attracted the attention of industry players. Presently, many network equipment manufacturers offer OpenFlow API on their commercial switches. Furthermore, because of the SDN’s popularity, many enterprises, such as Microsoft, Facebook, Google, Deutsche Telekom, and Verizon, have begun to support the Open Networking Foundation (ONF) [7] by endorsing SDN technology. However, typical of newly introduced technology, security vulnerabilities slowly surface as the SDN architecture becomes more popular and widely adopted. For example, SDN is vulnerable to DDoS attacks that exhaust the network resources, particularly the SDN controller’s bandwidth, processing, and memory, resulting in performance degradation or total disruption [8]. The following subsections discuss the motivation behind this SLR in Section 1.1, related works to this SLR in Section 1.2, and contributions and organizations of this SLR in Section 1.3.

### 1.1. Motivation behind This SLR

The motivation behind conducting an SLR on ML, DL, and hybrid-based approaches to detect and mitigate DDoS attacks on SDN networks is to provide an exhaustive overview of the existing research studies in this context and pinpoint the strengths and weaknesses of these approaches. Moreover, DDoS attacks are significant threats to SDN networks. However, traditional defense approaches may be ineffective in detecting and mitigating these attacks since attackers nowadays use new techniques to flood the SDN networks with different traffic variations (i.e., high and low rates), leading to the degrading of the SDN controller and making it unavailable to legitimate individuals [9].

ML and DL techniques have also been proposed as potential solutions to classify such attacks. Those techniques are employed to analyze the network traffic-flow patterns and detect abnormal traffic behaviors indicating DDoS attacks. However, there is a lack of consensus on the most effective ML, DL, and hybrid approaches to detecting DDoS attacks. Therefore, it aims to highlight these research gaps in the literature by systematically reviewing and synthesizing the existing approaches. By conducting an SLR, the authors provide a rigorous and transparent overview of relevant studies by identifying the key challenges and limitations of the existing approaches. This could assist the research society in determining sufficient techniques for detecting DDoS in SDN networks and developing a more robust and effective detection approach against such attacks.

### 1.2. Related Works

Many reviews discussed the impact of DDoS attacks on SDN networks and defensive security approaches, including several SLRs on SDN DDoS attacks. However, to the best of our knowledge, no attempt has been made to analyze, synthesize, organize, and structure systematically the existing studies on ML, DL, and hybrid approaches (combination of ML and DL algorithms) used in detecting and mitigating SDN DDoS attacks.

We performed a qualitative comparison with the existing SLR to highlight the distinctiveness of our work using several metrics, as tabulated in Table 1, determined by thoroughly investigating various existing detection approaches and surveys. The candidate’s surveys selected for comparison must fulfill the following criteria: (i) SLR related to SDN DDoS attacks; (ii) discuss the SDN architecture model; (iii) demonstrate an example of the OpenFlow forwarding process; (iv) provide an example of DDoS attacks on SDN networks; (v) provide online database sources for searching and retrieving the related studies; (vi) classify DDoS attack detection methods and limitations for ML, DL, and hybrid approaches; (vii) define the time span of published studies that coverage by the SLR (coverage years); and (viii) highlight the SDN datasets used.

To shed light on current related works in this context, Singh et al. [8] surveyed DDoS attack detection and mitigation approaches based on information theory, artificial neural networks (ANN), ML, and other related techniques. In addition, the authors discuss the SDN network architecture, the OpenFlow forwarding process, and the SDN DDoS attacks thoroughly. Moreover, Kaur et al. [10] contributed an SLR covering multiple DDoS security defense approaches at various locations within the SDN network architecture, including the control and data planes and the communication channel. In addition, it provides a comprehensive background on SDN architecture and summarizes many DDoS defense approaches in SDN networks, including ML-based ones.

Dalmazo et al. [11] focused on SDN and programmable network approaches, including a brief review of ML-based defense approaches in SDN. Meanwhile, Alashhab et al. [12] proposed a comprehensive survey of different ML approaches that are designed to protect SDN networks against low-rate DDoS attacks on SDN. Moreover, Alhijawi et al. [13] conducted a systematic review that classified the techniques based on detecting, mitigating, and preventing DoS and DDoS attacks against SDN controllers and SDN switches or based the SDN characteristics that use the SDN technology as a solution to handle DoS attacks in other network environments (i.e., traditional network, data center, and cloud computing).

Furthermore, Ali et al. [14] provided a systematic review of ML and DL-based approaches to detect DDoS attacks on SDN networks. In this SLR, the authors analyzed the most relevant studies based on ML and DL, then highlighted the strengths and weaknesses of these approaches. They also discussed the datasets, preprocessing strategies, evaluation metrics, experimental setups, and hyperparameters used in the literature. In addition, this thoroughly identifies the research gaps and future directions.

Overall, as shown in Table 1, our SLR study is distinctive qualitatively compared to other studies in SDN DDoS-attack detection and mitigation approaches. It provides a holistic overview of state-of-the-art approaches by reviewing and analyzing ML, DL, and hybrid approaches. Additionally, this SLR identifies the limitations, highlights open research problem gaps, and highlights the specific SDN realistic benchmark datasets that are publicly available.

### 1.3. Contributions and Organization of SLR

Research in SDN technology is still in its early stages of development. As a result, the SDN network is vulnerable to DDoS attacks. Despite this, several security approaches based on ML, DL, and hybrid already exist to detect such attacks. However, academicians and the security community need a clear view of the state and trend of the research in this area, which require additional effort to systematically review, synthesize, and perform an in-depth investigation of existing approaches. Therefore, we contribute an SLR that uses a systematic review methodology to explore those approaches thoroughly. In addition, this SLR collects and synthesizes all optimally selected studies to achieve the main objectives of this SLR. Overall, the contributions of this SLR are summarized as follows:

- Provides a solid background of the SDN architecture, underlines the forwarding process, and illustrates examples of SDN DDoS attacks.
- Presents theoretical background and practical steps that could be a reference or guideline for the research community to conduct SLR in any field.
- Presents new taxonomy of current research directions of state-of-the-art approaches for detecting DDoS attacks in the SDN network.
- A comprehensive review through critical analysis of 70 studies published from 2014 to 2022 on the existing literature of ML, DL, and hybrid approaches (incorporating both ML and DL) for detecting and mitigating DDoS attacks in SDN networks. It also underlines their limitations and highlights potential research gaps.
- Provides a critical analysis of the evaluation metrics, network simulators, hacking tools, experimental platforms, traffic analyzers, and up-to-date datasets used in the existing literature or studies related to detecting and mitigating SDN DDoS attacks.
- A list of challenges, open issues, and future research directions as a roadmap for researchers working on SDN DDoS attack detection approaches are given.

The remainder of this SLR is structured as follows. Section 2 presents the background of the SDN architecture model, illustrates an example of the OpenFlow forwarding process, and discusses DDoS attacks against SDN networks. Section 3 presents the research methodology of this SLR, while Section 4 underlines the results of this SLR. Section 5 demonstrates the research questions (RQs) results and discussion. Finally, the conclusions and limitations of this SLR are presented in Section 6.

## 2. Background

This section discusses the SDN architecture model, including explanations of the application, control, and data planes. Next, it explains the OpenFlow forwarding process, then underlines the DDoS attacks on SDN networks. Finally, this section discusses ML and DL-based DDoS detection approaches.

### 2.1. Architecture Model of SDN

**Figure 1:**
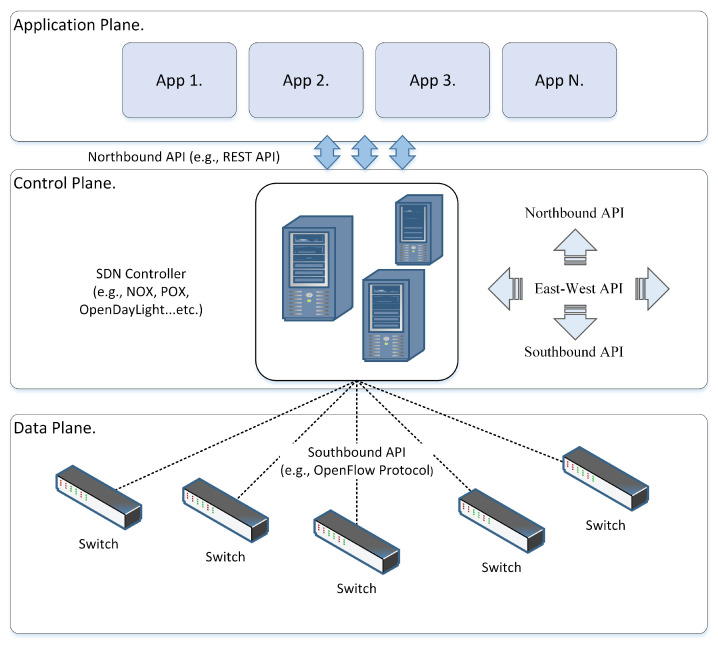
SDN architecture model in planes.

The SDN architecture emerged to address the complexity of traditional networks by enabling the creation of a scalable and adaptable programmable network [15]. It is the most significant technological revolution in the networking field in the past few decades. The architecture model’s central premise is the isolation of the data forwarding plane from the logical control of network devices. The SDN architecture model comprises three interconnected layers (application, control, and data layers) and two interfaces (southbound and northbound interface APIs), as illustrated in Figure 1. This segmentation results in an uncomplicated network that eases its manageability [16].

Figure 1 illustrates the SDN architecture’s three layers, each with a unique function and a distinct purpose. Some features are mandatory in SDN implementation, such as network operating systems (NOS), applications networks, southbound API, and northbound API, while others, such as language-based virtualization or hypervisor, are optional [17]. The following subsections underline all layers and interface APIs from top to bottom.

#### 2.1.1. Application Plane

The application plane resides on the upper layer of the SDN architecture model. This layer manages and configures the data plane network devices through a northbound API interface. In the meantime, it obtains network information from the control layer. The application layer includes many applications (software programs) that developers can quickly develop. This layer consists of six types of applications: (1) traffic engineering apps; (2) network analysis and monitoring apps; (3) fail-over apps; (4) network maintenance apps; (5) network security apps; and (6) different apps, such as firewall, prevention, and detection systems [18]. These applications define the functionality of the forwarding devices. An SDN device may perform multiple functions, unlike in a traditional network where each device is limited to performing only a single function [10] due to its static nature.

#### 2.1.2. Northbound API

The SDN architecture consists of two API interfaces: southbound and northbound, which play vital roles in connecting different layers. The northbound API is a communication interface between network applications operating in the application and control layers. The southbound interface primarily utilizes an open standard protocol (OpenFlow), while the northbound interface is an open standards protocol [19]. Meanwhile, due to advanced technology, various organizations and enterprises have introduced northbound interfaces (APIs), such as the RESTful API [20]. This interface is supported by most SDN controller platforms (i.e., [21] and NOX [22]), whereas other controller manufacturers introduce and define their own northbound API (i.e., Floodlight [23] and OpenDaylight [24]).

#### 2.1.3. Control Plane

The control plane is an intermediate layer between the application and data planes. It contains the NOS. It is also known as the SDN controller, which rules the entire network functionality and makes decisions on forwarding flows and dropping packets using programming [8]. It is an elemental plane in the SDN architecture where the complication is present. Fundamentally, the logical controller architecture is grounded on two ideologies: SDN controller objectives and SDN controller interfaces.

**Figure 2:**
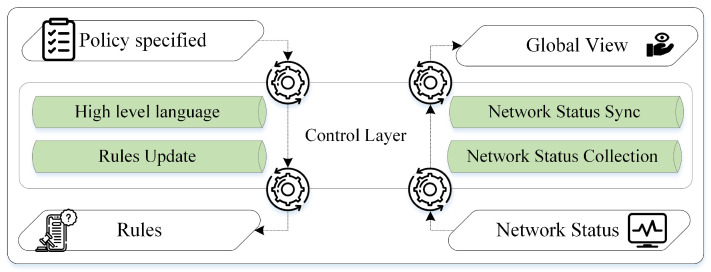
Logical design of SDN controller [25].

The SDN controller has two key goals: first, to control the network using the rules specified by the application plane and sending them to the infrastructure layer, and second, to monitor global and local network status. As a result, the controller contains two counter-reversing info flows, as shown in Figure 2. In the downward flow, the SDN controller interprets the application layer policies into forwarding packet rules. The critical feature of this procedure is to guarantee that the forwarding rules are valid and consistent. Meanwhile, in the upward flow, the SDN controller syncs network status gathered from the infrastructure layer to create a global view of the entire network and provide critical information (i.e., network topology) to the application plane for making network decisions [25].

Furthermore, various SDN controller interfaces, such as northbound, southbound, and east–west, are deployed to communicate with other planes and controllers. For example, the controller uses the southbound interface to cope with the infrastructure layer transactions, i.e., to update packet forwarding rules at switch devices at the infrastructure plane and gather network status. Moreover, the controller employs the northbound interface to interact with the application plane, i.e., to obtain the imposed policies from the application plane formatted in high-level languages and offer a synchronized global view [25]. At the same time, eastbound–westbound interfaces are deployed in the case of multiple controllers. Hence, the roles of these interfaces include sending and receiving data between controllers, verifying whether the other controller is up, and informing the other controller to control an asset of forwarding devices, which are essential for connecting additional controllers and planes [17,26].

Additionally, the control layer significantly impacts the performance of SDN networks, which relies on the scalability of the SDN controller. Controllers are involved in every transaction in the SDN network. When the initial packet of each flow arrives, switching devices must reactively seek matching forwarding rules from the controllers. Frequent communication between controllers and switching devices is paramount for rule updates and network status gathering. In this regard, bandwidth consumption and connection latency significantly affect the scalability of the control layer. Researchers suggested distributed controllers to overcome this scalability issue to enhance their performance, improve their processing capacity, and reduce the number of handled requests [25,27].

Since the architecture of the SDN controller platform can be centralized or distributed, a single SDN controller platform could handle all network switching devices in a centralized environment. However, although a centralized controller is an excellent benefit of the SDN network and sufficient to manage a small- to large-scale network, it is also a single point of failure if targeted by DDoS attacks [28]. However, in a distributed architecture, multiple SDN controllers can manage a cluster of nodes or a physically distributed group of elements. The controllers could be distributed at different sites or across the network. Each manages a network part to overcome the effect of a single controller failure [29]. These distributed controllers are efficient for large-scale networks such as data centers [17]. Indeed, the distributed controllers are logically centralized and physically distributed in the SDN architecture. In recent years, many SDN controllers have been highlighted, i.e., POX [21], NOX [22], Floodlight [23], OpenDaylight [24], and ONOS-distributed SDN [30].

#### 2.1.4. Southbound API

The SDN physically decouples the forwarding plane functions from the control logic plane. With the assistance of southbound API interfaces, the forwarding plane is kept on network devices, whereas the control plane is moved to an independent controller. Thus, southbound interfaces play a significant role in linking the control plane with the data plane. This link must stay accessible and secure; otherwise, the forwarding functions will not work [31]. The fundamental purpose of the southbound interface is to be used by the control plane to send management and monitoring messages to the data plane.

In contrast, the data plane bundles messages and sends them to the controller to report the current network status [18]. The southbound interface is commonly highlighted with the OpenFlow protocol [5] and touted as the standard interface defined by ONF [7]. The OpenFlow protocol is integrated with a secure communication protocol for secure communication through a southbound interface. Overall, this protocol is not mandatory, as the ONF suggests, since there are other protocols available, such as OvSDB (Open vSwitch Database) [32], OpenState [33], and OpFlex [34].

#### 2.1.5. Data Plane

The data plane is the lowest layer in the SDN architecture model. It is also called the infrastructure layer and comprises a collection of network devices (i.e., switches and routers) connected to form a robust network. Those devices are simple forwarding elements without the control logic to make any decisions because the data plane devices are running without network intelligence, which has been moved to an independent control system (i.e., SDN controller). It is essential to realize that these innovative network devices are constructed based on open and standard interfaces (OpenFlow), which guarantee standard configuration, intercommunication consistency, and compatibility across various data and control plane devices. Another critical point is that these open-standard interfaces allow the controller systems to effectively program different forwarding devices, which is problematic in conventional networks because of the heterogeneity of devices with proprietary and closed standards and the dispersed structure of the control plane [17].

**Figure 3:**
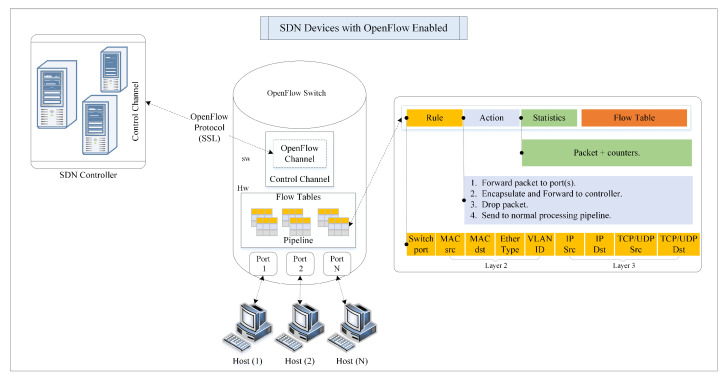
SDN devices with OpenFlow enabled.

Figure 3 shows that the SDN and OpenFlow architecture has two critical parts: controllers and forwarding devices. The controller is the network brain that runs on a commodity hardware platform. In contrast, the forwarding device for forwarding network packets can be in the form of software or hardware. Meanwhile, the OpenFlow device contains a pipeline of flow tables with three key elements: matching rules, actions, and counters. The match rule field includes several matching header fields, i.e., TCP or UDP, IP, Ethernet, and many others, depending on the OpenFlow protocol version. For example, OpenFlow version 1.0 and 1.5 contains 12 and 44 matching rule fields, respectively. Next, the actions describe the operation executed on the flow traffic, such as dropping, forwarding, sending to typical processing pipeline flow tables, and forwarding to the controller. Finally, the counter is for keeping track of the statistics of packets in every flow [10,17].

Additionally, there is at least one flow table and secure OpenFlow channel in an OpenFlow switch to ensure secure communication with the controller [35]. A pipeline between a series of flow tables describes how packets should be treated inside the OpenFlow device flow tables. Once a new packet is received, the pipeline table matching procedure begins with the first table and ends once a matching flow table is found [17]. However, if no matching flow table exists, the switch will act based on the table miss flow entry and report to the controller. The controller generates a new forwarding rule addressing the network state and sends it back to the switch. As soon as the OpenFlow switch receives these rules, it handles the subsequent packets by itself and forwards them accordingly [36]. OpenFlow is widely used on SDN data plane devices because of its simplicity and high-level paradigm.

### 2.2. OpenFlow Forwarding Process

The SDN controller is responsible for forwarding processing rules of the flows in the network by installing flow entries onto the OpenFlow-enabled switches [37]. The OpenFlow specification permits network switches to operate in proactive or reactive mode. In proactive mode, the backup flow rules are installed into the switches’ flow tables before receiving any network flow entries. The significant benefits of a proactive flow are a minimal setup time and reduced frequency of contacting the SDN controller. However, because SDN switches memory is a limited and expensive resource, preinstalling all backup rules in advance is not cost-efficient.

**Figure 4:**
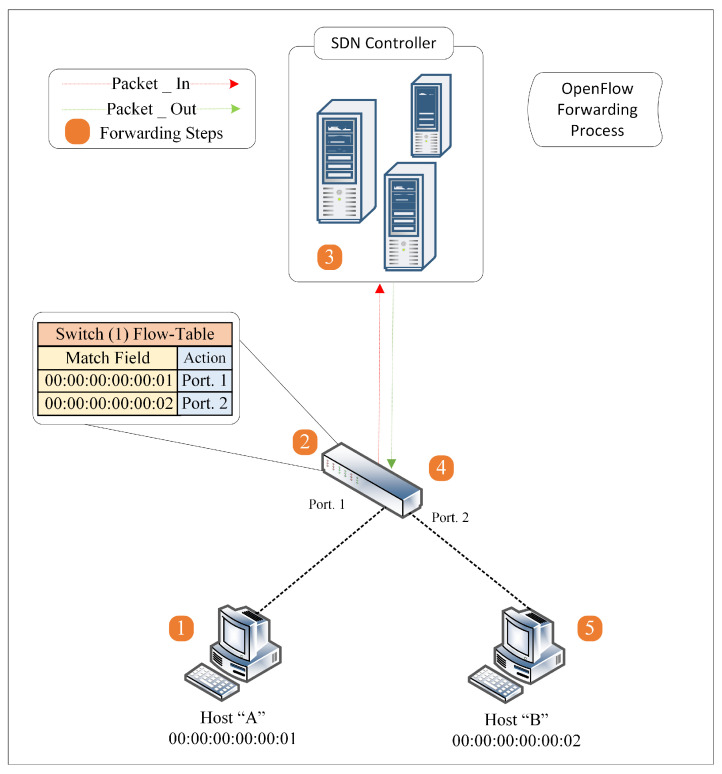
OpenFlow forwarding process.

In addition, because the backup rules are only utilized in case of failures, preinstalling all backup rules wastes the flow tables unnecessarily. Compared to the reactive mode, more events and interactions are involved, such as requesting, seeking, or computing the necessary rules. As packets arrive, the flow entry rules are dynamically installed on the switch, and this set of procedures is time-consuming and could cause packet delay [36]. This procedure is called the OpenFlow Forwarding process [1] and is illustrated in Figure 4 as a series of steps.

Figure 4 shows an example of an OpenFlow network comprising a controller, an OpenFlow switch, and two hosts, “A” and “B”. First, the OpenFlow switch initiates a TCP session. Then the controller detects the OpenFlow switch and starts the connection setup. The connection setup is via an OpenFlow secure channel over TCP, which the controller uses to manage the switch [38]. Likewise, hosts “A” and “B” connect to the OpenFlow switch via ports 1 and 2, respectively. In this example, the switch’s flow table is empty, and the switch does not know how to forward packets from source host “A” to destination host “B”. Under these circumstances, the OpenFlow forwarding process follows the following steps.

1. To deliver a packet to destination host “B”, source host “A” sends a packet to the SDN switch through port 1.
2. Upon receiving the packet, the switch performs a lookup in its flow tables. Suppose there is a match, then the switch will execute the instructions associated with the specific flow entry. Otherwise, the default OpenFlow switch specification (preinstalled OpenFlow rules) states that the packet must be forwarded to the SDN controller over the OpenFlow (southbound API) secure channel using a Packet-In message. The Packet-In message may include the complete packet or just a segment. In another case, the switch buffers the entire packet, and the Packet-In message contains a buffer ID of the packet [39].
3. The controller receives a Packet-In message from the switch. Typically, two things can happen here. First, the controller examines the packet header and checks whether it needs to send a Packet-Out message or not. If it does, include the reference buffer ID in the Packet-In message and the instruction for action to be performed (e.g., forward to the destination or drop the packet). Second, the controller might also send a flow modification message (Flow-Mod message) to the switch to install a new flow entry rule in the flow table to handle a similar subsequent flow in the future [31,39].
4. The switch receives the Packet-Out message from the controller, updates the flow table with a new flow entry, and then forwards the packet to the destination host “B”.
5. Finally, the destination host “B” receives the packet from source host “A”. When host “B” sends a return packet to host “A”, the reverse path of this communication follows the same steps.

A final note on this example: the switch no longer needs to forward packets to the SDN controller in subsequent communication between hosts “A” and “B” unless the OpenFlow switch’s flow entries timed out and expired or the network topology changed. This forwarding process is known as reactive mode, where the controller reactively installs the rules in response to Packet-In messages. However, reactively populating the flow rules in the flow tables exposes the switches to potential DDoS attacks. For instance, an attacker can easily take advantage of the reactive mode by sending massive Packet-In messages from the switches targeting the SDN controller, leading to network congestion and DDoS attacks [1,40].

### 2.3. DDoS Attacks against SDN Network

The control plane plays many essential roles within the SDN networks, including monitoring all network devices via secure OpenFlow channels, configuring flow tables, and providing instructions or rules to switches to manage new traffic flows. In addition, the controller may oversee the whole network by assuming the manager’s role between the data plane and application plane via southbound, northbound, and east/westbound API interfaces in the case of distributed controllers. Centralizing the controller simplifies the network operations by having a global view of the entire network. In addition, the controller uses the network traffic flow statistics as a baseline input (information) to an attack detection technique to determine whether the network flow is normal or abnormal. As a result, the controller is critical in any endeavor to improve and enhance the SDN security productivity against potential attacks. Hence, it must become a highly targeted point for significant attacks and malicious behaviors [41].

Additionally, the SDN controller is the most complicated part of the network because of logical centralization, and it is responsible for implementing forwarding rules for every new flow entry. This complexity increases security threats, especially when overwhelmed by a massive number of incoming spoofed packets or flows that instantly result in poor controller performance and lead to a bottleneck. As a result, the controller is very vulnerable to DoS and DDoS attacks, one of the most challenging and damaging network threats [8]. Consequently, many attackers target the SDN controller to make it unavailable. Unfortunately, not even multiple controllers can offer a practical solution to such attacks because they only increase the complexity due to additional efforts required to maintain the consistency, authentication, and scalability of many forwarding policy rules in each domain. In addition, multiple controllers need extra secure communication channels to communicate. However, there are no standard specifications for the communication protocol and its security for controllers to transmit state information. As a result, the inter-controller communication channels may become a target, eventually leading to cascading failure for other controllers [42].

On top of that, DDoS attacks are easy to execute but challenging to detect and mitigate. Cyber attacks frequently utilize a botnet, or a network of computers, to conduct successful DDoS attacks. The most common DDoS attacks evade detection and increase the possibility of reaching targeted victims by employing several attack scenarios and various DDoS attack tactics. Depending on the attack protocol level, DDoS attacks can be classified into two groups. First, DDoS flooding attacks at the transport/network layer. These attacks often leverage TCP, UDP, ICMP, and DNS packets to disrupt legitimate individual connectivity by draining the entire SDN network bandwidth capacity. Second, application-layer DDoS flooding attacks disrupt online services by exhausting the resources of the server that hosts the corresponding services [43].

An attacker can quickly launch SDN DDoS attacks by flooding the SDN controller with spoofed UDP, ICMP, or TCP packets to cripple it, rendering it out of service. The strength of a DDoS attack lies in its destructive impact on the victims, and the effect could multiply by employing various attack scenarios upon the target. Attackers using DDoS attacks usually exploit compromised hosts to launch attacks to avoid detection. First, the attacker scans the network for vulnerable hosts to exploit. Once compromised, the attacker uses the compromised hosts to send malicious network traffic with forged source IP addresses toward one or more targeted victims in the SDN network.

In addition using compromised hosts to launch DDoS attacks, attackers also employ other methods to avoid detection, such as using fewer packets that consume less bandwidth, mimicking the normal traffic behavior, and varying the attack traffic rates. Attackers vary attack traffic rates by mixing low-rate and high-rate DDoS attacks, which confuses the majority of DDoS attack detection mechanisms. In addition to avoiding detection, those methods also increase the efficacy of the attacks [44,45].

**Figure 5:**
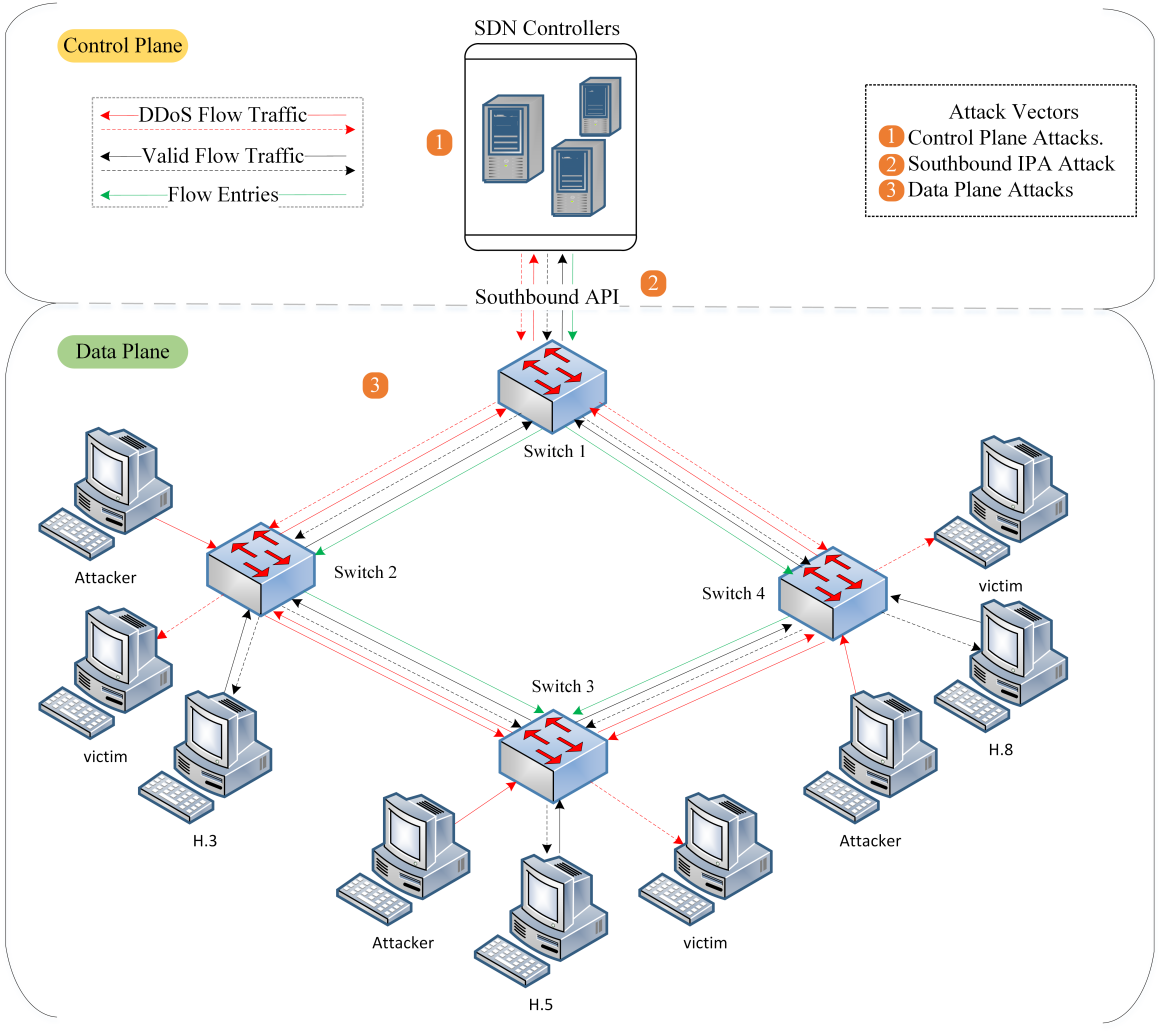
OpenFlow forwarding process.

Since attackers spoof attack packets’ source IPs, the OpenFlow switches will fail to find a matching rule for the incoming malicious packets, resulting in the packets being forwarded to the SDN controller and eventually exhausting its resources. Then the SDN controller becomes unreachable for the subsequent incoming packets. Even with a backup controller, it ultimately will suffer the same fate of cascading failure [46]. Therefore, protecting the SDN controller efficiently against low-rate and high-rate DDoS attacks is crucial. Figure 5 illustrates the DDoS attack mechanism on the SDN network.

Furthermore, DDoS attacks pose critical threats to the SDN network, especially if the controller becomes a target, directly or indirectly [9]. For example, when a switch receives invalid network packets, it buffers all packets and then forwards only the packets’ headers to the controller using Packet-In messages. However, once the switch’s memory is full, a buffer overflow will occur at all switches in the data plane, causing heavy congestion that impacts the entire data plane switches and the secure OpenFlow control channels (Southbound API). Therefore, the DDoS attack victims are not just the SDN controller but also the network switches and the southbound IPA that connects the switch and the SDN controller. The network switches will experience a buffer overflow attack, and the southbound IPA will suffer a bandwidth saturation attack. Therefore, DDoS attacks on the SDN controller will fall under a global impact attack category since they could cause failure to the entire network [1].

Ultimately, the SDN design might increase network security threats by providing programmability properties to assist modern intelligent systems, such as intrusion-prevention systems (IPS) and intrusion-detection systems (IDS). However, even though the importance of SDN characteristics in enhancing network security has been underlined, the techniques for securing the SDN controller have not been adequately addressed, leaving the network vulnerable to attacks. For example, conventional DDoS attack detection methods are inefficient because conventional networks differ from SDN network environments. In addition, the DDoS attacks against SDN controllers may be readily carried out using low-cost hacking tools and do not necessitate high-performance computation or much effort from the attackers [41].

### 2.4. ML and DL-Based Approaches

Artificial intelligence (AI) is a broad term for techniques that let dummy machines mimic the human brain or intelligence to solve real-world problems using ML and DL algorithms [47]. ML is a subclass of AI that contains various algorithms that enable a machine to learn systematically by running mathematical models to detect and classify SDN DDoS attacks. Some examples of the most commonly used ML algorithms in IDS are the K-nearest neighbor (K-NN), support vector machine (SVM), decision tree, ANN, K-mean clustering, and fast learning network [48].

ML algorithms are known as shallow learning algorithms, as opposed to DL, a subclass of ML that incorporates multiple invisible layers to obtain deep network properties. Because of their deep and critical construction to learn the relevant features of the dataset all by itself and provide an output, those algorithms outperform the ML techniques. Some examples of the standard DL algorithms are recurrent neural networks (RNN), convolutional neural networks (CNN), deep belief networks (DBF), deep neural networks (DNN), deep Boltzmann machine, and the autoencoder [49].

Moreover, the ML-DL consists of algorithms that operate on autonomous learning capability and predict the final results. For example, IDS can use these algorithms to protect SDN networks against multiple threats, including DDoS attacks. ML-DL algorithms workflow learns from a dataset during the model training stage. The IDS-based ML-DL algorithms can be categorized as supervised, unsupervised, and semi-supervised. The IDS model discriminates between normal and malicious traffic patterns using supervised ML algorithms by giving a collection of network flows labeled before training. However, manually labeling each dataset is time-consuming [50]. Moreover, the ML-DL consists of algorithms that operate on autonomous learning capability and predict the final results. For example, IDS can use these algorithms to protect SDN networks against multiple threats, including DDoS attacks. ML-DL algorithms workflow learns from a dataset during the model training stage [51]. The IDS-based ML-DL algorithms can be categorized as supervised, unsupervised, and semi-supervised. The IDS model discriminates between normal and malicious traffic patterns using supervised ML algorithms by giving a collection of network flows labeled before training. However, manual labeling of each dataset is time-consuming [50].

In addition, the unsupervised ML-DL techniques extract critical information or features directly from the unlabeled dataset. At the same time, semi-supervised learning trains the model by using the labeled and unlabeled dataset. According to earlier research, supervised ML-DL techniques outperform unsupervised ML-DL techniques in IDS efficiency. Further, as has been noticed, the ML and DL models, either supervised or unsupervised, have been used recently in ensemble methods to benefit from various classifiers, since some algorithms perform well for detecting a particular type of attack and others show poor performance on different kinds of attacks. The ensemble technique combines weak classifiers through training many classifiers and then forming the more robust classifier by voting [49].

## 3. Research Methodology

This paper conducts an SLR to classify studies relevant to the research area or answer specific research questions related to SDN DDoS attack detection approaches. The SLR is the most suitable and dependable method to document and evaluate existing research studies. The SLR approach allows researchers to summarize the strengths and weaknesses of the existing research studies, conduct a comprehensive investigation to identify potential research gaps and future trends and challenges, and contribute a solid framework and background to establish a new research area.

The SLR protocol followed in this research is based on the Kitchenham and Charters guidelines for conducting SLR in software engineering [52,53]. This SLR methodology consists of three main parts: planning, conducting, and reporting. Each part involves distinct stages, and those stages are: defining the review protocol, Section 3.1, defining research questions, Section 3.2, qualifying conditions (Inclusion and Exclusion Criteria), Section 3.3, search plan, Section 3.4, research studies selection procedure, Section 3.5, quality assessment (QA) criteria, Section 3.6, and Data extraction and Data synthesis, Section 3.7. The details of each stage will be discussed in the following subsections.

### 3.1. Defining the Review Protocol

The methods utilized to conduct a systematic review are specified in the review protocol to eliminate the risk of researcher bias. Therefore, a preplanned methodology is required. Without a procedure, it is conceivable that the researchers’ expectations would influence the selection of studies or the analysis. This would result in investigations being left out of the sample studies required to conduct a thorough examination and gain a broad knowledge of the phenomena. The review protocol identifies the research questions, search plan, study selection procedures, quality assessment, and data extraction and synthesis [52].

### 3.2. Defining Research Questions (RQ)

Fundamentally, defining the research question(s) is vital in the planning strategy to build a robust SLR protocol. The research question formulation demands a significant review of literature studies. The main objective of this SLR is to conduct a comprehensive review of the existing approaches for the detection and mitigation of SDN DDoS attacks. In addition, to highlight the research findings and demonstrate the valuable outcomes clearly and achieve these objectives, the following research questions are defined:

- **RQ(1)**. What are the existing ML-based approaches to detect and mitigate DDoS attacks against SDN networks?
- **RQ(2)**. What are the existing DL-based approaches to detect and mitigate DDoS attacks on SDN networks?
- **RQ(3)**. What are the existing hybrid-based approaches to detect and mitigate DDoS attacks on SDN networks?
- **RQ(4)**. What evaluation metrics, network simulators, hacking tools, and experimental platforms are used in the literature studies?
- **RQ(5)**. What datasets are used to evaluate and validate the existing approaches, and are there any publicly available realistic datasets for DDoS attacks on SDN networks?
- **RQ(6)**. What are the challenges, open-issue perspectives, and future research directions for DDoS attacks on SDN networks?

This SLR formulated six in-depth research questions, demonstrating each question’s motivation as follows. RQ(1), RQ(2), and RQ(3) contribute to the comprehensive exploration of different types of existing detection and mitigation approaches (based on ML, DL, or hybrid) on SDN DDoS attacks. Meanwhile, RQ(4) identifies the prevalent evaluation metrics, network simulators, hacking tools, and experimental platforms used in existing literature studies. RQ(5) contributes to determining the characteristics of the used datasets to evaluate the current approaches. Finally, RQ(6) contributes to underlining DDoS attacks’ challenges and future research directions in securing SDN networks from DDoS attacks. The following subsection elaborates on the inclusion and exclusion criteria used for this SLR.

### 3.3. Inclusion and Exclusion Criteria Terms

The SLR protocol must define the inclusion and exclusion criteria terms to ensure that the selected studies are related to the study area and answer the defined research questions. The main reason for setting up the criteria is to ensure that the included studies are relevant and related to ML, DL, and hybrid approaches (a combination of ML and DL algorithms) to detect and mitigate SDN DDoS attacks. Therefore, the selected studies must match all the predefined criteria terms. Table 2 tabulates the inclusion and exclusion criteria terms of this SLR.

In addition, the studies that do not meet the inclusion criteria (in Table 2) are excluded. Moreover, a screening operation is conducted to select the relevant literature studies related to this review context. The screening operation has three stages:

(i) **Title and abstract stage**: this stage excluded the irrelevant studies based on title and abstract. Next, the studies that meet at least some criteria terms in Table 2 are selected and passed to the next stage for further processing.
(ii) **Full-text reading stage**: this stage excluded the studies based on full-text or partial reading if they did not meet the criteria terms in Table 2.
(iii) **Final selection stage**: This stage applies the criteria terms in Table 2 for final selection and excludes studies that do not match any of the following criteria.
  - The research study must be relevant and related to the research questions.
  - The research study discusses the comprehensive solution ML, DL, and hybrid-based approaches to detect and mitigate DDoS attacks on SDN networks.
  - The research study provides a sufficient volume of technical implementation and methodology information.
  - The research study presents an adequate description of the obtained results.

### 3.4. Search Plan

The search plan involves querying several digital databases, as shown in Table 3. The databases, *Scopus, IEEE Xplore, Taylor & Francis Online, Science Direct, Web of Science (WOS), Wiley Online Library, Springer Link, and ACM Digital Library*, cover almost all impact factor journals, magazines, and relevant conference proceedings to this SLR.

In this stage, an advanced search was performed using Boolean OR/AND operators to link the keywords, terms, synonyms, and abbreviations. The search plan consists of two main stages, automatic and manual search. The first stage involves performing an automatic search using the predefined keywords based on the research questions of this SLR. For example, the following keywords are used: *(“Software Defined Networking” AND “Distributed Denial-of-Service”) OR (“SDN” AND “DDoS”) AND (“Intrusion Detection System” OR “IDS” AND “Network Security”)*. Those keywords are derived from the defined research questions and the structure of this SLR to cover the most relevant and related studies.

In addition, the search string is created and explored in the digital database sources by utilizing predefined keywords. Furthermore, the search string is stored on all database sources to ensure we receive a notification of every newly published article after completing the search. Once retrieved from the database sources, the acquired research papers are chosen according to the research questions and inclusion and exclusion criteria. The second stage involves manual screening of the primary studies’ references using backward and forward search techniques to track the primary studies’ citations. We utilized Google Scholar to move forward to discover relevant studies cited by the primary studies, identifying any missing ones (from the first stage) to ensure this SLR is sufficiently comprehensive [54]. Therefore, after every primary study’s references are reviewed and the inclusion and exclusion criteria applied, the obtained studies from this stage are added to Mendeley. Mendeley helps us to efficiently manage the collected studies and quickly remove duplicate studies to develop a final set of selected studies.

### 3.5. Research Study Selection Procedure

The research study selection procedure plays a vital role in identifying the research studies that are related to the research questions of this SLR. Using the automatic search technique identified 1968 studies. Section 3.4 describes the predefined keywords used to retrieve relevant research studies from every database source. According to [55], the retrieved studies must go through several stages to ensure that the excluded studies are unrelated to this SLR topic.

**Figure 6:**
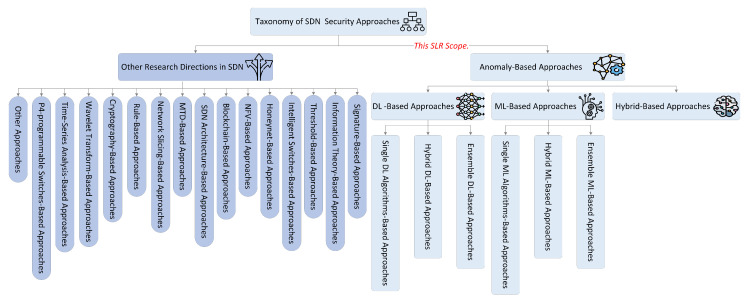
Taxonomy of existing approaches for detection of DDoS attacks in SDN networks.

The first stage excludes duplicate research studies using Mendeley references manager, resulting in 1489 studies. The second stage, applying the inclusion and exclusion criteria terms (as presented in Table 2), extracts the relevant studies and excludes the irrelevant and unrelated ones, resulting in 613 studies. The third stage, applying the inclusion and exclusion criteria based on the title and abstract, resulted in a total of 260 studies. Simultaneously, from those studies, we provide a new taxonomy of state-of-the-art approaches for detecting SDN DDoS attacks, as shown in Figure 6.

In addition to this stage, after retrieving studies from the predefined online digital databases, a notification alert was configured on every digital database to notify us of any newly published articles, which resulted in 13 studies. Then, those studies count towards the third stage for screening titles and abstracts, and the selected studies are passed to the subsequent stage. The fourth stage involves employing the inclusion and exclusion criteria based on a full-text reading of the studies. It then makes the decision to include or exclude the selected studies, resulting in 68 studies.

**Figure 7:**
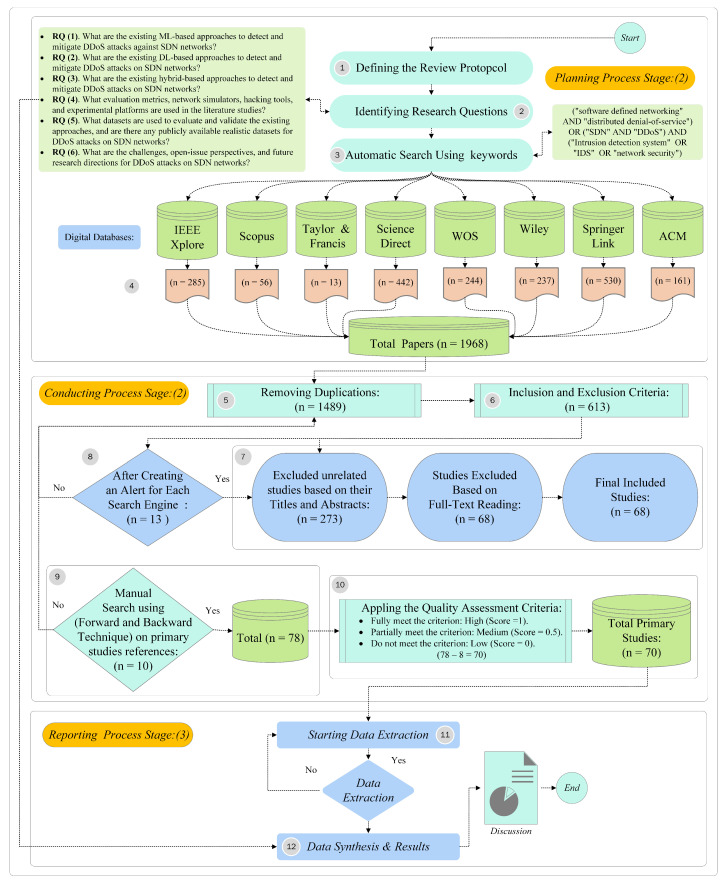
Overall research methodology protocol.

The final stage involves manual search using forward and backward techniques on the primary study’s references to trace any relevant missing studies. The search finds an additional ten relevant studies missed in the previous stage. Therefore, the comprehensive studies resulted in a total of 78 studies. However, after the full-text reading stage to evaluate the studies’ quality, 8 studies were excluded according to the inclusion and exclusion criteria (see Table 2) and quality assessment (QA) criteria. Therefore, the total number of studies recognized as primary studies is 70, which served as the foundation of the QA criteria. Figure 7 illustrates a flow diagram of the selected study’s procedure and presents the overall research methodology.

### 3.6. Quality Assessment (QA) Criteria

A QA generally uses instruments such as a checklist of factors or several questions to evaluate primary studies. In addition, the main reason for applying QA is to judge the quality of the selected studies [56,57]. Therefore, this SLR defines nine quality assessment criteria to evaluate the quality of every study, as listed in Table 4, inspired by [58], covering all the research study characteristics, including design, conduct, analysis, and conclusion.

The QA procedure determines a study’s level of quality based on nine quality assessment criteria to check the credibility of 70 primary studies. The study’s quality is determined by whether it scored high, medium, or low on each quality criterion listed in Table 4. Consequently, the studies that fulfill the criterion have a score of 1. If a study partially meets the standard, a score of 0.5 is given. However, studies that do not meet any criterion will score 0 points. Therefore, studies with a total score greater than or equal to 6 will be considered high-quality. Studies with a score between 5 and 5.5 will be considered medium-quality. In contrast, studies with a total score of 4 to 4.5 will be considered low-quality studies. Finally, we excluded studies with a score of 3.5 or lower since they failed to meet the criteria for inclusion and exclusion (refer to Table 2).

### 3.7. Data Extraction and Data Synthesis

This stage underlines the procedure of data extraction and synthesis by carefully reading all 70 selected studies and abstracting and saving the related data using Microsoft Excel spreadsheets plus the Mendeley reference manager. This stage creates a form of data-extraction items and reports all information collected from primary studies [52]. Therefore, this SLR considered the following columns: *Study ID, bibliographic info (title, author, publication source, and publication year), type of publication, study objective(s), method used, datasets type, evaluation metrics, study finding, study limitations, and study experiment.* In addition, this review selected those items following the objectives and research questions. Table 5 shows the form used to extract data items for the 70 studies. In addition, as soon as the data is extracted from the primary studies and recorded to identify the final view of the SLR results, the analysis stage is processed using descriptive synthesis. The following section explains the synthesis results.

## 4. SLR Results

Before discussing the data analysis of the SLR, this section gives significant statistical results of the primary studies in terms of publication sources and timeline, study characteristics, and quality assessment results. The following section will discuss and go through this SLR data synthesis analysis.

### 4.1. Sources and Year of Publication

**Figure 8:**
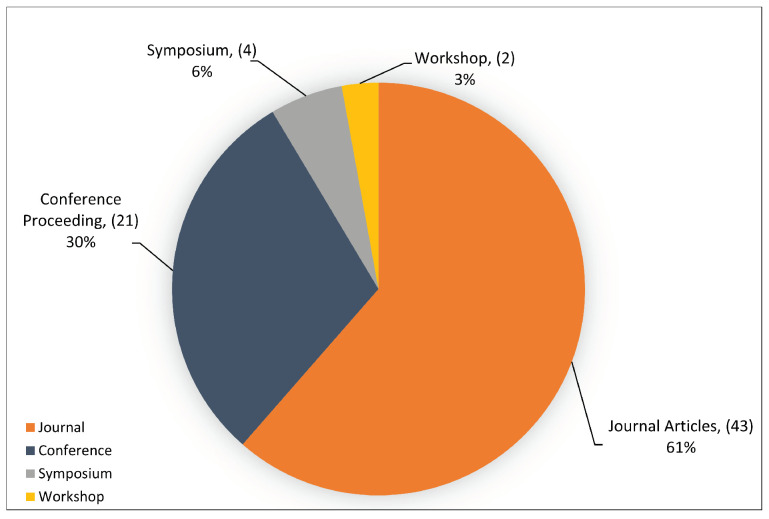
Distribution of primary studies by sources of publications.

The distribution of the primary studies based on their publication sources is depicted in Figure 8. It can be seen that the majority of the studies were published in scientific journals, with a percentage of 61%, which equates to 43 primary studies. Meanwhile, 21 studies were published at conferences proceeding, with 30%. In addition, 4 studies were published in symposiums, with a percentage of 6%, and 2 studies were published in workshops, with 3% of the total primary studies. Thus, most primary studies were published in scientific journals, which increases the SLR’s reputation and overall quality assessment criteria.

**Figure 9:**
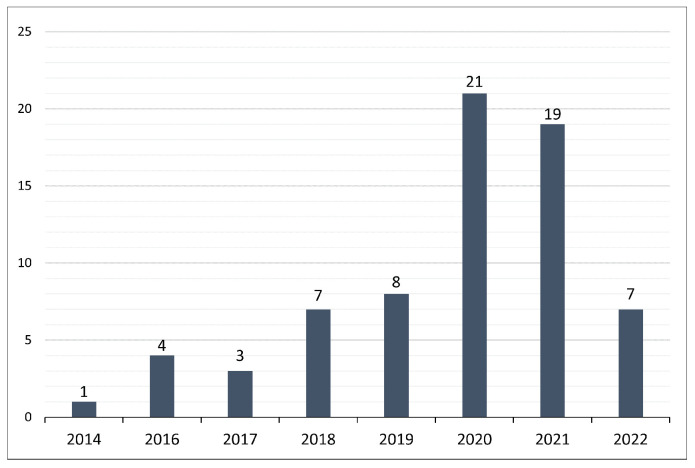
Distribution of primary studies by year of publications.

Furthermore, the primary study period of this SLR is from 2014 to 2022, as shown in Figure 9. Additionally, Figure 9 illustrates the distribution of the primary studies over eight years. The progressive growth in the number of publications related to DDoS attacks on the SDN network since 2014 shows a steady increase, particularly from 2016 onward. Furthermore, Figure 9 shows that most of the studies were published in 2020, with 21 studies, and then in 2021, with 19 studies. We find this surprising, since SDN DDoS attacks have only been around for a few years.

### 4.2. Primary Studies’ Methods

**Figure 10:**
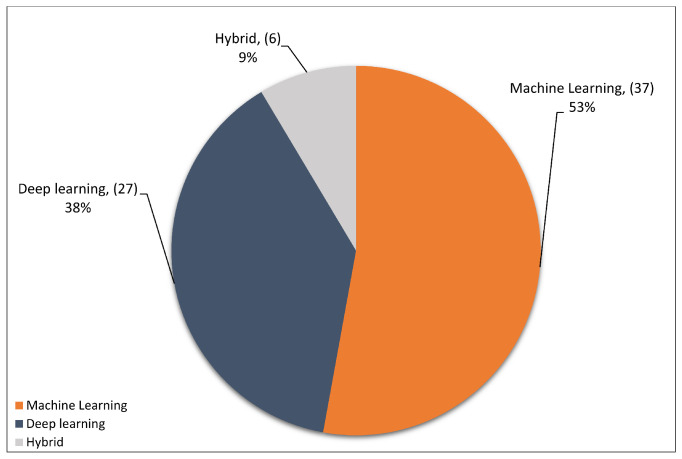
Distribution of primary studies based on methodology.

This section highlights the most frequent methodologies of primary studies. The selected studies are based on ML, DL, or hybrid approaches for detecting DDoS attacks in the SDN network. Figure 10 shows the distribution of primary studies based on methodology. As can be seen, most selected studies were based on ML techniques, with a percentage of 53%, equating to 37 primary studies. Meanwhile, 27 studies were based on DL techniques, accounting for 38% of the selected studies. The remaining six studies, with a percentage of 9%, were based on a hybrid approach that combines machine and DL techniques, which is considered the minority of the primary studies. Overall, ML and DL methods have been regularly used, and in a few studies, both ML and DL methodologies are integrated to complement each other.

### 4.3. Quality Assessment Results

**Figure 11:**
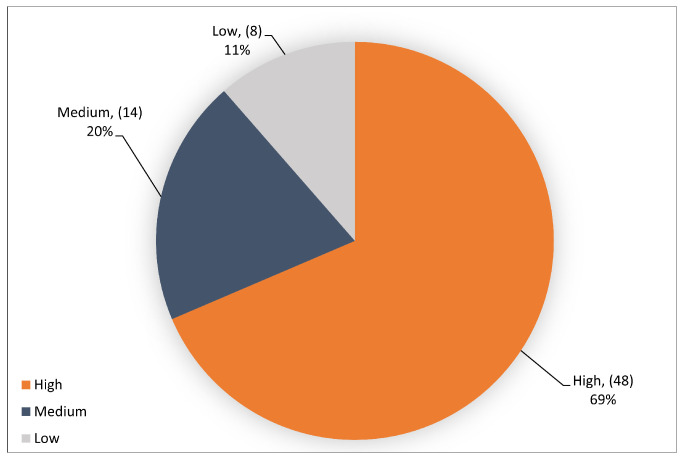
Distribution of primary studies based on QA levels.

The quality assessment has been performed as subjectively as possible. However, despite the possibility of subjectivity, we feel that the quality assessment gave us a general sense of the included studies. Therefore, this SLR evaluated every empirical study based on the nine criteria described in Section 3.6 Table 4. During this process, 8 studies did not fulfill the inclusion and exclusion criterion, so they were excluded from the list. Thus, based on QA criteria, the primary studies of this SLR included 70 articles, and the majority of the remaining studies got a relatively high-level score. Figure 11 shows the results of the quality assessment score levels. However, most of the studies scored at a high level, equating to 48 primary studies, which is sufficient to make this SLR a valuable contribution. In comparison, medium and low levels correlated to 14 and 8 of the primary studies, respectively. The results of utilizing the quality criteria are stated in Table A1.

## 5. Research Questions Results and Discussion

Generally, IDS protects the network by detecting traffic to determine any adversarial attacks in the network. This SLR focuses on anomaly-based IDS based on ML and DL techniques or integrated (also called hybrid approaches) to increase the detection accuracy of DDoS attacks on SDN networks. Thus, this SLR scope on ML, DL, and hybrid-based approaches to detect and mitigate DDoS attacks against the control plane, data plane, and secure communication channel of the data-control plane in the SDN network. Therefore, this section discusses and answers the RQs (as stated in Section 3.2) based on the selected (70) primary studies and their analyses. The RQs are discussed in the following subsections.

### 5.1. RQ(1). What Are the Existing ML-Based Approaches to Detect and Mitigate DDoS Attacks against SDN Networks?

This section presents a comprehensive overview of ML-based approaches for detecting and mitigating DoS and DDoS attacks against SDN networks. Three different ML approaches are classified based on the methodology: first, the enhancement of ML approaches through the ensemble technique of multiple ML algorithms to improve the overall performance, particularly the detection accuracy; second, the development of ML-based approaches through hybridization or relying on multiple ML algorithms; and third, techniques that investigate a single ML classification algorithm. This section also underlines the essential findings of every study, followed by a summary table that tabulates ML approaches with their limitations. Finally, these approaches are thoroughly discussed as follows.

#### 5.1.1. Ensemble ML Approaches

Ensemble approaches might include several types of classifiers, and the ensemble itself may consist of different ML classifiers. The training process may also be concluded by combining several independent classifiers. For example, Ref. [59] propose an optimized weighted voting ensemble (OWVE) model to detect and mitigate DDoS attacks. The ensemble model uses SVN, random forests (RF), and gradient-boosted machine classifiers with different hyperparameter values. The ensemble model shows high classification accuracy of 99.41% and 99.35% for CIC-DDoS-2019 and CAIDA-2007 datasets. Ref. [60] proposed an ensemble ML model called the voting-based intrusion detection framework for protecting SDN against DDoS attacks. The proposed voting model was trained and tested using three datasets: UNSW-NB15, CICIDS2017, and NSL-KDD, and achieved better detection accuracy than other approaches.

Ref. [61] developed an ensemble ML method called K-mean and RF to improve the accuracy and efficiency of classifying and detecting DDoS attacks. The proposed system was tested and trained with the InSDN dataset and achieved a perfect detection accuracy (100%). Finally, an ensemble ML based on K-NN, naïve Bayes (NB), SVM, and self-organizing map (SOM) algorithms to detect abnormal behavior was proposed by [62]. The approach uses CAIDA 2016 dataset for testing and triaging the model. However, the ensemble approach achieved low detection accuracy and false-positive rates for ensemble and single ML algorithms.

#### 5.1.2. Hybrid ML Approaches

Several hybrid ML-based approaches, such as [63], combined SVM and random forest (RF) classification algorithms to classify normal and DDoS attack traffic. The approach was tested and evaluated with a realistic SDN dataset, achieving high accuracy (98.8%|) and few false alarms. Ref. [64] proposed a hybrid approach based on SVM and SOM to enhance the classification performance for detecting DDoS flooding attacks against OpenFlow switches and SDN controllers. The approach was tested on the CAIDA dataset, achieving 98.13% and 97.6% for detection rate and accuracy, respectively.

Ref. [65] investigated P4 programmable and K-NN, RF, SVM, and ANN algorithms for implementation in a real-time detection systems. They proposed an automated DDoS attack detection (DAD) method. The DAD approach detects SYN flood attacks locally on SDN switches with an overall performance of 98%. To protect the SDN controller against DDoS attacks, Ref. [66] employed RF, SVM, K-NN, naïve Bayes (NV), and decision tree (DT) algorithms. The approach was evaluated with the NSL-KDD dataset, achieving a high accuracy (99.97%) for DT but a very low accuracy (60.19%) for SVM.

Ref. [67] investigated a variety of ML classification models, such as DT, random forest (RF), AdaBoost (AB), multiayer perceptron (MLP), and logistic regression (LR), to analyze and detect TCP-SYN flood DDoS attacks against the SDN controller. The experiment results show that all classification models achieved high performance. Ref. [68] employed ML algorithms, such as K-NN, DT, ANN, and SVM, to classify SDN network traffic as normal or DDoS attacks. They showed that DT has the best accuracy rate (99.75%) among classification algorithms, and the worst is the SVM algorithm with 81.48%.

Ref. [69] proposed a detection and classification model based on ML. They adopted four popular classifiers (i.e., K-NN, quadratic discriminant analysis (QDA), Gaussian naïve Bayes (GNB), and classification and regression tree (CART)) to detect TCP, UDP, and HTTP flood DDoS attacks. CART outperforms others in average training time (12.4 ms), prediction accuracy (98%), and prediction speed. Ref. [70] proposed a detection and mitigation approach against DDoS attacks. They utilized an SVM as the primary classifier, followed by kernel principal component analysis (KPCA) for feature selection strategy and a Genetic Algorithm (GA) for enhancing the parameters of the SVM. The proposed model achieved a detection accuracy of 98.907%.

In addition, different approaches rely on multiple ML algorithms, such as [71]. They used six machine-learning algorithms (NB, SVM, K-NN, extreme gradient boosting (XGBoost), DT, and RF) to protect an SDN network from DDoS attacks. The XGBooSt algorithm obtains the highest accuracy (99.7%), and the rest achieve low accuracy. Ref. [72] proposed a detection approach based on DT and SVM algorithms for detecting DDoS attacks. The proposed approach was tested and evaluated using the KDD CUP dataset. However, they only achieved low performance. For example, DT and SVM have accuracy rates of just 78% and 85%, respectively.

Ref. [73] use four ML classification algorithms (i.e., KNN, SVM, ANN, and NB) to detect DDoS attacks in an SDN environment. The proposed approaches were evaluated with a synthetic dataset, achieving a high detection accuracy (98.3%) for KNN in detecting DDoS attacks. In contrast, the rest of the ML classifiers only achieved a relatively low detection accuracy. [74] proposed a flexible IDS to detect and mitigate low-rate SDN DDoS attacks. They train the IDS with six ML algorithms (i.e., RT, REP tree, RF, SVM, MLP, and J48), then evaluated using the CIC-DoS-2017 dataset. The proposed IDS achieved a moderate detection rate performance of 95%.

Ref. [75] proposed a framework based on K-Means and K-NN algorithms to detect DDoS attacks. They utilized the data plane switches to deploy a detection trigger mechanism to reduce the controller’s overhead. The framework was evaluated with synthetic and NSL-KDD datasets, and both achieved high detection accuracy. Ref. [76] proposed a DDoS attack mitigation scheme for the SDN network based on a bandwidth-control mechanism and the extreme gradient boosting (XGBooST) algorithm for precise attack detection and optimal network resource utilization. They validated the scheme in an SDN environment and achieved 99.9% accuracy and a low false-positive rate. In addition, the proposed system runs on the controller.

Ref. [77] proposed an approach inspired by the human body’s immune system called the “Artificial Immune System-IDS” (AIS-IDS). The proposed approach utilizes biologically inspired fuzzy logic that automates network anomaly detection and mitigation. The system was evaluated with synthetic and CICDDoS 2019 datasets, surpassing other classifiers in detection accuracy and other performance metrics. In addition, the proposed approach was implemented on the SDN controller. Ref. [78] proposed an approach based on SVM, DT, NB, and logistic regression (LR) to detect DoS and DDoS attacks in the SDN network. The approach was evaluated with a synthetic dataset and achieved an accuracy of 97.5% for SVM, 96% for NB and DT, and 89.98% for LR.

Ref. [79] proposed an approach based on SVM, DT, K-NN, and BN classifiers for detecting DDoS attacks. The approach was tested and trained with the NSL-KDD dataset, showing that no classifier achieved a higher detection rate than the DT classifier (95.16%). Ref. [80] employed MLP, RF, SVM, and DT algorithms to detect TCP SYN and UDP flood DDoS attacks against the SDN controller and flow-table switch and bandwidth saturation attacks. The proposed model was tested and trained with a synthetic dataset, showing that the controller DDoS attack has the lowest classification results (less than 90% accuracy for SVM and MLP) than flow-table switch and bandwidth attacks. Ref. [81] proposed a system defense based on SVM, J48, and NB algorithms for detecting DDoS attacks. The proposed defense system was trained and tested on the NSL dataset and had a detection accuracy of 99.40% for classification.

Ref. [82] proposed a rapid SDN defensive system against DDoS and port scan attacks by analyzing IP flow traffic every five seconds. The proposed system runs on the SDN controller and uses particle swarm optimization (PSO), MLP, and discrete wavelet transform (DWT) for anomaly detection. In addition, it mitigates DDoS attacks by using a game-theoretical approach. The proposed defense system performance is functioning for detecting DDoS and port scan attacks, and the mitigation techniques successfully restored the SDN to its previous state. Finally, Ref. [83] proposed an approach based on seven ML algorithms (i.e., K-NN, RF, NB, SVM, linear regression (LR) DT, and ANN) to classify and detect DDoS (i.e., HTTP, UDP flooding attacks, and Smurf). The proposed approach is implemented at the SDN controller and achieves high average detection accuracy for all classification algorithms.

#### 5.1.3. Single ML Approaches

Several approaches employ a single ML algorithm to detect and mitigate DDoS attacks, such as [84]. The proposed approach uses an RF to classify normal and abnormal traffic based on the flow of entries. The packets classified as a DDoS attack will be mitigated by implementing specific switches’ rules. As a result, the detection system detects DDoS attacks with an overall accuracy of 98.38%, with the shortest mitigation time. Ref. [85] proposed an Advanced-SVM algorithm for detecting UDP and SYN flood DDoS attacks in SDN networks. The proposed system was tested and trained using SDN-TrafficsDS and KDDCUP99 datasets, achieving overall average evaluation performance of 87%, 84%, and 93% for precision, recall, and F1-score, respectively.

Ref. [86] proposed a detection approach based on the factorization machines (FM) to detect low-rate DDoS attacks against SDN data planes with improved accuracy. Although the method works well at detecting low-rate DDoS attacks on SDN data planes, the proposed approach achieved a relatively moderate detection accuracy at 95.80%. Ref. [87] proposed an approach in the SDN controller to detect UDP and SYN flood DDoS attacks on SDN networks by employing an ASVM algorithm. The proposed approach was tested and trained using a synthetic dataset, achieving 97% detection accuracy and a 2% false-alarm rate.

Ref. [88] proposed an approach based on SVM to detect SDN DDoS attacks. The approach was evaluated with the DARPS dataset, achieving 95.11% accuracy and a low false-positive rate. Wang et al. [89] proposed a safety guard scheme (SGS) on SDN switches to protect the SDN control plane from DDoS attacks. The proposed system employs the back-propagation neural networks (BPNN) technique for anomaly detection. The proposed scheme can detect and respond to DDoS attacks directly.

Ref. [90] proposed an approach based on the fast K-NN model because of its efficiency and accuracy in detecting DDoS attacks. The proposed method was evaluated and trained on an unrealistic NSL-KDD dataset. As a result, the proposed approach enhanced the K-NN detection efficiency in detecting DDoS attacks, achieving high accuracy, precision, and stability. Another study by [91] proposed a method based on an improved K-NN algorithm that runs on the SDN controller for detecting DDoS attacks, which also achieved high performance in detecting DDoS attacks.

Ref. [92] proposed an approach for detecting and mitigating ICMP, SYN flood, and UDP flood DDoS attacks. Based on the traffic flow classifier, they used a BPNN for online DDoS detection and evaluated their model with a synthetic dataset. The proposed approach shows a moderated detection accuracy of 96.13%. Ref. [93] proposed an approach based on an SVM to detect DDoS attacks. The approach was tested and trained using the KDD-99 dataset, preserving 99.8% detection accuracy.

Ref. [94] employed a distributed self-organizing map (DSOM) with OpenFlow switches to combat the flooding attacks and address other issues such as alleviating performance bottlenecks of the communication channel and reducing controller overhead. The OpenFlow switches carry the security modules, and the application layer handles each module running within the OpenFlow switches. The DSOM efficiently detects abnormal traffic and reduces computation overhead. Ref. [95] proposed an approach named “Software-Defined Anti-DDoS” (SD-Anti-DDoS) to reduce the heavy workload of the SDN controller and switches. This approach relies on the BPNN technique to classify normal and abnormal traffic flow. As a result, the SD-Anti-DDoS approach can respond to DDoS attacks and reduce the controller’s workload. Table 6 provides an overview of the ensemble, hybrid, and single-ML-based approaches, including their limitations.

In summary, Table 6 shows that most approaches in the literature fall under the hybrid category, followed by single- then ensemble-ML category. Furthermore, most researchers use self-generated realistic datasets to evaluate and train their proposed approach, while few others resort to publicly available unrealistic datasets due to the lack of benchmark datasets for SDN DDoS attacks. In addition, most studies use feature-selection techniques to select the optimum features to improve the detection accuracy and classification of network traffic. However, some studies did not use them, such as [66,91]. At the same time, most studies ran their approaches on the SDN controller, adding unnecessary overhead to the controller, such as [70,76,82,83,84].

In addition, some researchers run their approaches out of the SDN controller to reduce the load and overhead, mainly during DDoS attacks, such as [74,95]. In contrast, some studies did not provide details about where they deploy their approaches, such as [80,85,88]. Moreover, most approaches are designed to detect or mitigate DDoS attacks, and only a few can do both [83,92]. In addition, most ML approaches are limited to detecting or mitigating high-rate DDoS attacks [63,68,75], which are achievable with high accuracy due to the availability of a large amount of malicious traffic. However, only a few ML approaches can detect low-rate DDoS attacks in SDN networks, such as [67,74,86]. Overall, most ML-based approaches achieve high detection accuracy, such as [59,61,63,69].

### 5.2. RQ(2). What Are the Existing DL-Based Approaches to Detect and Mitigate DDoS Attacks on SDN Networks?

This section discusses ensemble, hybrid, and single DL techniques for detecting SDN DDoS attacks. Once again, this section highlights the key findings of each research, followed by a table that summarizes the key parameters and their limitations. The following subsections underline DL techniques.

#### 5.2.1. Ensemble DL Approaches

Ref. [96] used CNN, gated recurrent unity (GRU), and long-short-term memory (LSTM) for classifying DDoS attacks. The proposed model was trained with the CICIDS 2017 dataset and achieved 99.77% detection accuracy in the case of a small number of features. Ref. [97] proposed an IDS based on DL ensemble techniques, using CNN, RNN, and DNN for detecting DDoS attacks. The ensemble model trained with the CICIDS2017 dataset achieved a high detection accuracy of 99.05%. Another CNN-based ensemble approach to detect SDN DDoS attacks was proposed by [98]. The ensemble model was evaluated with the ISCX 2012 dataset and achieved 98.48% detection accuracy.

#### 5.2.2. Hybrid DL Approaches

Ref. [99] utilize CNN and LSTM for detecting traffic anomalies caused by DDoS attacks. The proposed approach was evaluated using the InSDN dataset and achieved detection accuracy of 96.32%. Ref. [100] proposed a hybrid DL model running on the SDN controller. They employ the CNN and LSTM DL algorithms for early detection of DDoS attacks. The hybrid approach was evaluated with the CICIDS2017 dataset and achieved a high detection accuracy of 99.45%. Ref. [101] proposed an approach implemented on the SDN controller by employing CNN and LSTM for detecting low-rate SDN DDoS attacks. The proposed approach was evaluated with a synthetic dataset and achieved a high performance of more than 99%. [102] proposed a system implemented on the SDN controller by deploying an RNN, a GRU, and an LSTM to protect the SDN controller against DDoS attacks. They evaluated their proposed approach using the InSDN dataset and achieved a high detection accuracy.

Ref. [103] proposed a method to protect the SDN controller against DDoS attacks after investigating many classifiers, such as LSTM and CNN. The proposed approach was evaluated with the synthetic dataset and achieved a low detection accuracy of 89.63%. Ref. [104] propose an approach based on RNN with an autoencoder for detecting SDN DDoS attacks. The proposed method was evaluated using the CICDDoS-2019 dataset and achieved a high detection accuracy of 99% compared to other ML techniques. Ref. [105] proposed a DL-based IDS (DeepIDS) to detect zero-day DDoS attacks. They employ the DNN and gated recurrent neural network (GRU-RNN) algorithms. The proposed system was evaluated using the NSL-KDD dataset and achieved low detection accuracy of 80.7% and 90% for DNN and GRU-RNN, respectively.

Ref. [106] resorted to RNN and GRU to improve the detection rates of their previous and current anomaly-based IDS for SDN networks. They used NSL-KDD and CICIDS2017 datasets for training, testing, and evaluating their proposed approach, achieving an accuracy of 89% for the NSL-KDD dataset and 99% for the CICIDS2017 dataset to detect DDoS attacks. Ref. [107] proposed a detection approach based on CNN, RNN, and LSTM algorithms to detect DDoS attacks. The proposed approach was evaluated using ISCX 2012 dataset. The verification accuracy of the proposed model for detecting DDoS attacks is 98% for test data and 99% for training data.

Ref. [108] proposed an IDS based on GRU and RNN (GRU-RNN) to classify SDN network traffic, whether normal or anomalous (attack). Unfortunately, the detection rate of the proposed approach achieved a relatively low detection accuracy of 89% and 90% for normal and attack traffic, respectively. Ref. [109] proposed a two-level DDoS attack detection in SDN networks using entropy to detect spoofed switch ports and CNN as a classifier to increase accuracy and efficiency and reduce training costs. As a result, the proposed approach achieved a high accuracy of 98.98% for the DL model, but low accuracy of 92.37% and 96.97% for information entropy and the two-level method, respectively. Ref. [110] proposed a hybrid approach based on CNN and a transformer (composed of an encoder and a decoder) to detect DDoS attacks. The proposed approach was tested on the CICDDoS2019 dataset and achieved the highest performance compared to other approaches.

#### 5.2.3. Single DL Approaches

Several approaches employ a single DL algorithm, such as [111], who proposed a controller-based security system to detect various attacks as early as possible. The proposed approach was trained and tested using the InSDN dataset and achieved a high traffic classification accuracy and highly efficient latency and throughput performance. At the same time, Ref. [112] proposed a GRU-based SDN defensive system to detect DDoS attacks. The proposed system analyzes each IP flow traffic record to reduce the severity of the attack against SDN and enables quicker mitigation reactions. The approach was tested using two scenarios with two datasets (CICDDoS 2019 and CICIDS 2018), and both achieved high detection accuracy.

Ref. [113] proposed a detection and defense system implemented on the SDN controller. The system utilizes a generative adversarial network (GAN) to detect DDoS attacks. The proposed defense system was evaluated using a real SDN network dataset for the first scenario and CICDDoS 2019 dataset for the second scenario, achieving 99.78% and 95.54% DDoS attack detection rates, respectively. Ref. [114] employ stacked autoencoder multi-layer perceptron (SAE-MLP) algorithm to detect DDoS attacks. The proposed approach was tested and trained with a realistic SDN dataset and achieved a high detection accuracy of 99.75%.

Ref. [115] proposed an IDS based on the CNN algorithm to improve the detection of intrusions. The proposed approach was evaluated and tested with the InSDN dataset, achieving 93.01% detection accuracy. Makuvaza et al. [116] explored the use of DNN to detect SDN DDoS attacks. The proposed approach was evaluated with the CICIDS 2017 dataset and achieved 97.25% detection accuracy. Meanwhile, Ref. [117] investigated the bi-directional recurrent neural network (BRNN) algorithm for classifying SDN DDoS attacks. The proposed approach was trained and tested with a synthetic dataset and achieved 99.21% detection accuracy.

Ref. [118] proposed a real-time mitigation agent based on deep reinforcement learning to mitigate TCP, UDP, ICMP, and SYN flood DDoS attacks in the SDN network environment. As a result, around 85% of normal traffic reaches the server when the mitigation agent is operational. Arivudainambi et al. [119] proposed an IDS based on the lion optimization algorithm (LOA) for feature selection and CNN for DDoS attack classification. The proposed system was evaluated with the NSL-KDD dataset and achieved an overall classification accuracy of 98.2%. [120] proposed a network application system in the SDN controller to detect DDoS attacks against the control and data planes. The proposed approach employs the stacked autoencoder (SAE) to detect multi-vector DDoS attacks (i.e., TCP, UDP, and ICMP) in SDN network environments. As a result, the proposed system detects the DDoS attack classes with 95.65% accuracy.

Ref. [121] implemented a network intrusion detection system (NIDS) in the SDN controller to monitor network traffic flows. The proposed NIDS uses DNN to detect flow-based anomalies in SDN networks and classify the flow as normal or abnormal. However, their proposed NIDS achieved a low accuracy of 75.75% when evaluated using the NSL-KDD dataset. Meanwhile, Ref. [122] proposed a detection system for detecting SDN DDoS attacks that uses an unsupervised restricted Boltzmann machine algorithm. The proposed system was evaluated with a synthetic dataset and achieved 92% detection accuracy and 8% false-positive rate. Table 7 summarizes the DL-based approaches with their limitations.

Table 7 shows that most existing approaches fall under hybrid and single-DL categories, followed by ensemble DL. The most popular dataset used for evaluating and training proposed approaches is an unrealistic dataset. However, at the same time, many researchers resort to private synthetic datasets due to the lack of publicly available benchmark datasets. Furthermore, most studies use feature selection techniques to select the optimum features to improve the detection accuracy and classification of network traffic. However, a few studies, such as [96,103], did not use them.

As for the deployment location, most approaches run on the SDN controller, resulting in additional overhead to the controller. On the other hand, a few approaches [107,109] operate outside of the SDN controller, which avoids adding unnecessary load and overhead to the controller, especially during DDoS attacks. Unfortunately, some studies [98,110,114] lack details about the deployment location. Even though most approaches are designed only to detect DDoS attacks, a few can also mitigate them. In addition, most DL approaches are limited to detecting or mitigating high-rate DDoS attacks with high accuracy, which is feasible due to the abundance of abnormal traffic flow. Only one DL-based approach [101] can detect low-rate SDN DDoS attacks. As a result, most DL-based approaches achieve high detection accuracy for high-rate DDoS attacks but not for low-rate ones.

### 5.3. RQ(3). What Are the Existing Hybrid-Based Approaches to Detect and Mitigate DDoS Attacks on SDN Networks?

The literature reveals that a wide range of anomaly detection systems based on methods such as ML and DL algorithms have been assembled and used recently. However, several researchers combined ML and DL techniques to develop a hybrid approach. This subsection presents an overview of these hybrid approaches to detecting SDN DDoS attacks. Ref. [123] proposed a hybrid IDS by combining CNN with classical ML algorithms (i.e., SVM, KNN, and RF). The hybrid model was evaluated using InSDN, CSE-CIC-IDS2018, and UNSW-NB15 datasets, showing the approach can detect abnormalities even with a minimal number of a subset of 9 features. Compared to a single CNN, the combination of CNN, SVM, and, most importantly, the RF algorithm produces superior results.

Ref. [124] employed various ML algorithms (i.e., KNN, SVM, and RF) and DL (i.e., MLP, CNN, GRU, and LSTM) algorithms for detecting transport and application-layer DDoS attacks. The proposed approach was simulated in an SDN environment and achieved 98% and 95% detection accuracy for transport and application layer DDoS attacks, respectively. Ref. [125] proposed a hybrid model running on the SDN controller. The hybrid approach combines an autoencoder with a one-class SVM to detect DDoS attacks. The proposed model was evaluated with the CIC-IDS-2017 dataset and achieved a high average accuracy of 99.35%. In the meantime, Ref. [126] proposed an approach that runs on the SDN application plane to detect and mitigate DDoS and port-scanning attacks. They employed Shannon entropy, LSTM, and fuzzy logic algorithms and then evaluated them with two scenarios. The first scenario utilized a synthetic dataset, and the second CICDDoS 2019 dataset achieved high performance for the first scenario and satisfactory performance for the second one.

Additionally, a hybrid model proposed by [127] aimed for better classification outcomes with the lowest training time. They employed LSTM, autoencoder, and one-class SVM (OC-SVM) to detect potential attacks. The model was trained and tested using the InSDN dataset but only achieved a relatively low detection accuracy of 90.5%. Ref. [128] proposed a detection system that employs an SVM and a DNN to detect anomaly-based DDoS attacks, and they used the KDD CUP dataset for training and testing the model, achieving a low detection accuracy of 74.3% and 92.3% for SVM and DNN, respectively. Table 8 summarizes the hybrid-based approaches and their limitations.

Table 8 underlines the hybrid approaches that integrate the ML and DL algorithms in the final analysis. As can be seen, most studies trained their approaches with realistic datasets. In contrast, a few evaluated and trained their approaches with unrealistic datasets, such as [125,126]. In addition, most studies used feature selection techniques to select the optimum features to improve the detection accuracy and classification of normal or abnormal network traffic, except [126,128].

In addition, some studies [124,126,128] ran their approaches on the SDN controller, which added unnecessary overhead to the controller. However, some studies did not mention the deployment location of their approaches, such as [123,125,127]. Moreover, most approaches either detect or mitigate DDoS attacks, but not both, except [126]. In addition, all the hybrid-based approaches are designed for high-rate DDoS attacks, not low-rate ones. As a result, most hybrid approaches achieve high accuracy, such as [123,124,125,126], while some have low accuracy in detecting or mitigating SDN DDoS attacks, such as [127,128].

### 5.4. RQ(4). What Evaluation Metrics, Network Simulators, Hacking Tools, and Experimental Platforms Are Used in the Studies?

This RQ discussed the extracted data related to the evaluation metrics. Furthermore, this section underlines the experimental platforms researchers used to run their approaches. Finally, this section further explores the hacking tools and network simulators used in the literature studies.

#### 5.4.1. Network Simulator and Tools

**Figure 12:**
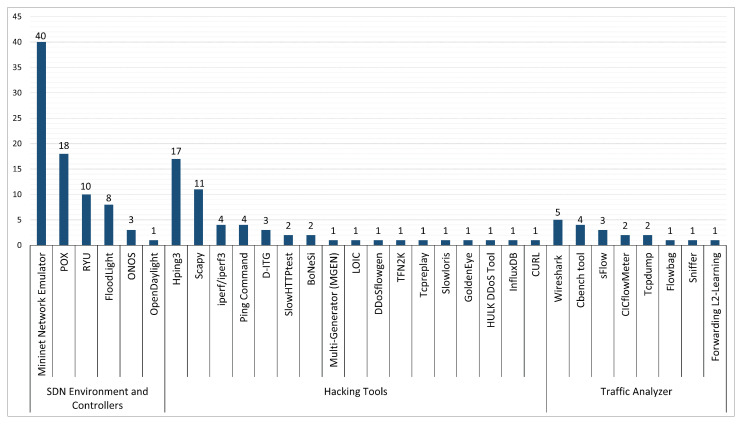
Distribution of using network simulator, hacking tools, and traffic analyzer.

Based on the data extracted from the literature studies in RQ Section 5.1–Section 5.3, this section analyzes the employed SDN network environment and SDN controllers, hacking tools, and network traffic analyzer. As for the used SDN network environment, it is clear from Figure 12 that most researchers utilize the Mininet network emulator to create their SDN testbed environment. Furthermore, most studies used the Python-based open-source POX controller in their network environment as their SDN controllers and Hping3 and Scapy as hacking tools to generate normal and malicious SDN DDoS traffic. Lastly, the Wireshark program is the most popular network traffic analyzer used to collect and filter network traffic for analysis.

#### 5.4.2. Experimental Platforms

**Figure 13:**
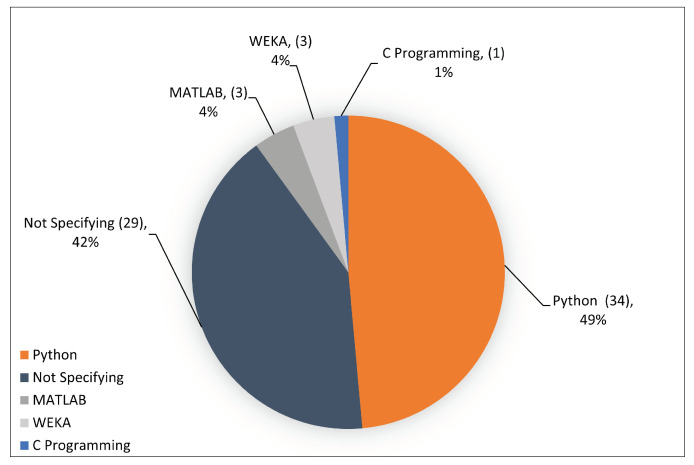
Distribution of experimental platforms.

The existing approaches are designed, implemented, and executed on different experimental platforms. Based on the literature studies in RQ Section 5.1–Section 5.3, four experimental platforms are the most frequently used platforms to design and implement their approaches. Figure 13 indicates that most existing studies implemented their approaches using the Python programming language with the help of well-known back-end libraries, such as Tensorflow and Keras. Those approaches make up 49%, representing 34 of the literature studies. Meanwhile, three studies used MATLAB, and three others used WEKA data mining tools to implement and execute their approaches. In addition, only one study used the C programming language for implementation. The remaining 29 studies that showed promising results in detecting SDN DDoS attacks did not specify the experimental platforms used in their implementation.

#### 5.4.3. Evaluation Metrics

**Figure 14:**
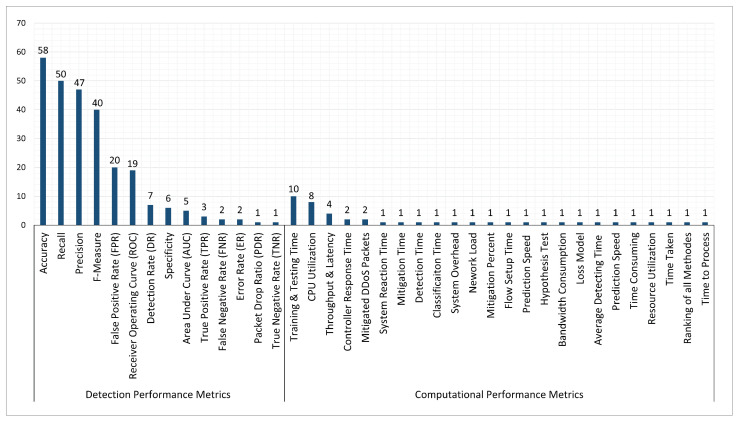
Distribution of evaluation metrics in the existing studies.

The evaluation metrics are extracted from literature studies in RQ Section 5.1–Section 5.3. As shown in Figure 14, there are two main classes of performance evaluation metrics: detection and computational. The detection performance metrics contain all measures commonly used by researchers to validate the results of their approaches. These measures are based on a confusion matrix used to evaluate the performance of classification algorithms (i.e., true positive (TP), true negative (NT), false positive (FP), and false negative (FN). In total, 14 performance matrices belong to this class and have been adopted by many existing studies.

Moreover, Figure 14 shows that accuracy is the most frequently used evaluation metric. It was used in 58 studies, followed by recall (50), precision (47), F-measure (40), false positive rate (FPR) (20), and receiver operating curve (ROC) (19). In addition, the detection rate, specificity, and area under curve (AUC) are only found in 7, 6, and 5 studies, respectively. In the meantime, only a small number of studies use other evaluation metrics, such as the true-positive rate (TPR), the false-negative rate (FNR), the error rate (ER), the packet drop ratio, and the true-negative rate (TNR).

The computational performance metrics measure the computational performance of the proposed approach. There are 24 different computational performance metrics pinpointed from the literature studies. For example, Figure 14 illustrates that ten studies evaluated their approaches based on taring and testing time. Then, CPU utilization, throughput and latency, controller response time, and mitigation of DDoS packets were examined in 8, 4, 2, and 2 studies, respectively.In addition, as stated in Figure 14, the remaining computational performance metrics were investigated by few studies, indicating that these metrics are unique computational performance metrics. In summary, it is recommended that the research community evaluate their approaches with various evaluation metrics rather than relying on one or three conventional evaluation metrics, which are no longer valid criteria for evaluating the contributed approaches.

### 5.5. RQ(5). What Datasets Are Used to Evaluate and Validate the Existing Approaches, and Are There Any Publicly Available Realistic Datasets for DDoS Attacks on SDN Networks?

**Figure 15:**
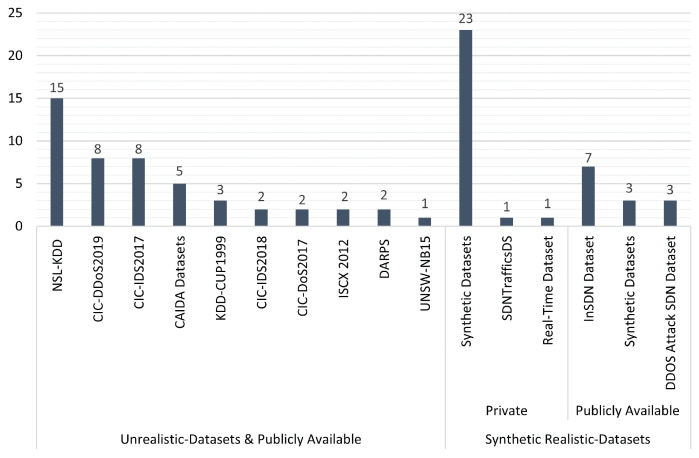
Distribution of datasets used in the existing studies.

The dataset types have been extracted based on literature studies in RQ Section 5.1–Section 5.3. Figure 15 shows the distribution of the datasets used to evaluate and validate the existing approaches. As can be seen, most studies used synthetic datasets. Typically, these datasets are self-generated datasets using a simulated network environment. Therefore, most researchers prefer to generate private datasets due to various reasons, such as difficulties in setting up real-world SDN networks, privacy concerns, and security issues. Another reason for resorting to a private dataset is the flexibility to evaluate their approaches in various scenarios. Finally, since there are no standards or criteria to set up the network environment and visualize the simulation scenarios, it is harder for researchers to compare their proposed approach with others [129].

Figure 15 shows two primary dataset types used by researchers. The first type is realistic datasets, which include synthetic, InSDN, DDOS attack SDN, real-time, and SDN TrafficsDS datasets. These datasets are considered realistic synthetic datasets that reflect the SDN network characteristics since they are explicitly made for SDN-based DDoS attacks. Three of those realistic synthetic datasets are publicly available: [126], InSDN [130], and DDOS attack SDN datasets [131]. The second type is unrealistic datasets, including the remaining datasets in Figure 15. Although publicly available, the benchmark datasets were not designed for SDN networks but for traditional networks.

### 5.6. RQ(6). What Are the Challenges, Open-Issue Perspectives, and Future Research Directions for DDoS Attacks on SDN Networks?

SDN technology enables better manageability and more flexibility to address the limitations of conventional networks. However, at the same time, it poses many security threats that must be investigated. Therefore, this section highlights the security challenges and open issues related to existing security approaches. Further, this section provides future research directions and gaps to be addressed. The implications for researchers and practitioners of security challenges and open-issue perspectives are discussed as follows.

#### 5.6.1. Security Challenges

The security challenges for researchers and practitioners are listed as follows:

- **DDoS attacks against SDN controllers:** This SLR argues that DDoS attacks against SDN controllers can be classified as “global impact” attacks since they could cause the failure of the entire network. Many approaches, such as [63,67,80,102,104,112,113,120], are designed to detect DDoS attacks on SDN controllers since the controller is an essential component of the SDN network, providing critical functionality and network management authority. It is responsible for coordinating and managing network traffic flows, implementing network configuration, and installing forwarding rules on the data plane devices. Consequently, it becomes an enticing target for attackers to attempt DDoS attacks. Thus, DDoS attacks against SDN controllers remain an open security challenge that needs consideration.
- **Availability of realistic datasets:** This SLR notes that most researchers used self-generated datasets using different hacking tools, network simulators, and various experimental platforms. At the same time, few researchers still rely on the existing unrealistic benchmark datasets, such as NSL-KDD, CIC-DDoS2019, and KDD-CUP1999, to train and evaluate their approaches, even though the datasets do not reflect the characteristics of an SDN network environment and do not represent flow-based SDN architecture adequately. Finally, some researchers contribute realistic synthetic datasets by publicly making them available [126,130,131]. The variety of datasets utilized results in the research community finding it challenging to compare their works with others [129]. Therefore, the availability of a realistic SDN dataset remains an open research challenge that needs addressing.
- **Distributed SDN controllers:** This SLR remarked that the security approaches proposed in most literature studies are based on a topology with a single network controller, such as [69,75,77,86,113]. However, this topology is vulnerable to single points of failure in the case of DDoS attacks. In contrast, a network with distributed controllers in a flat or hierarchical design is much more efficient in load distribution, consistency, and scalability. In addition, as the severity of DDoS attacks increases, these distributed controllers can maintain network efficiency whenever the central controller becomes a bottleneck. They can reduce the impact of DDoS attacks and communication overhead, eliminate single points of failure, and ease the load balancer’s traffic flow among multiple controllers. Therefore, the operation of distributed SDN controllers remains an open security challenge that needs further investigation.
- **High-rate and low-rate DDoS attacks:** This SLR highlights that most approaches in literature studies detect and mitigate high-rate DDoS attacks with high accuracy and a low false-positive rate. High-rate DDoS attacks are easy to predict due to a noticeable increase in the attack traffic volume in the flow. However, in recent years, a different type of DDoS attack has surfaced, known as low-rate or stealthy attacks, which are very challenging to detect and mitigate with high detection accuracy and low false-positive rates due to the similarity of attack traffic flow with legitimate network traffic flow. Some studies, such as [74,86], try to detect low-rate DDoS attacks, but only achieve low performance. Therefore, detecting and mitigating low-rate DDoS attacks with high accuracy and a low false-positive rate remains an open research challenge that needs addressing.
- **Deployment of detection approaches:** This SLR found that most literature studies deployed their approaches at the SDN controller, such as [82,91,102,103,104,106,111]. The main reason is that the forwarding switches send normal and abnormal traffic flows to the controller for attack detection or requesting new forwarding rules. Furthermore, the controller needs to regularly collect and monitor the network flow statistics, which could add communication overhead and interrupt the detection process. Typically, the SDN architecture experience overload while detecting and mitigating SDN DDoS attacks; therefore, an efficient approach must be implemented outside the SDN controller to address these challenges and reduce the controller’s overhead. If implemented this way, the proposed approach for detecting and mitigating SDN DDoS attacks must cooperate with the controller and the data plane switches.
- **Prevention approach for DDoS attacks:** This SLR demonstrated that most literature studies, such as [59,66,70,74,75,76,77,81,84,90,92,95,106,109,112,113,114,118,126] focus on detection and mitigation instead of prevention of SDN DDoS attacks. There is a lack of approaches concerning prevention besides the detection and mitigation approaches. Preventing DDoS attacks protects the SDN network’s functionality from deteriorating, which is more urgent than detecting and mitigating them by stopping their propagation into the network and consuming its resources. Therefore, DDoS attacks’ prevention, detection, and mitigation are still an open research challenge that needs addressing.
- **Feature-selection techniques and hyper-parameter tuning:** This SLR highlights that most literature studies, such as [61,67,77,87,95,97,102,109,122,125,127] use various feature selection techniques to select the most relevant network traffic features. A thorough feature selection process significantly enhances the ML-DL approach’s performance. Thus, feature selection techniques warrant further investigation using other strategies that could further improve ML and DL-based approaches. In addition, it is essential to hyper-tune the ML and DL models to obtain the best parameters for practical training and avoid negative impacts. Through hyper-parameters, the ML and DL models perform well when the hyper-parameters are tuned or optimized. Therefore, the feasibility of incorporating novel feature selection techniques and hyper-parameters to improve ML-DL-based detection, mitigation, or prevention of SDN DDoS attacks remains an open research challenge that needs addressing.

#### 5.6.2. Future Research Directions

Based on the findings of this SLR and its perspectives, the future research directions of DDoS attack approaches on SDN networks are listed as follows:

- **Towards DL-based approaches:** This SLR studied and analyzed the three most popular approaches (i.e., ML, DL, and hybrid) to detect and mitigate SDN DDoS attacks. However, the trend is shifting towards the DL-based approach according to the amount of interest it has generated within the research community in recent years. The most crucial benefit of DL over classic ML is higher performance in analyzing massive datasets. Moreover, several technologies, such as cloud computing and IoT systems, have adopted SDN technology to handle enormous amounts of data that needs to be processed [132]. Hence, DL-based approaches and techniques naturally fit into SDN and emerging technologies since their computational architecture already includes many processing layers that can train data at different grades of complexity. Therefore, the outcomes or new findings in DL-based research have a promising future for incorporation into SDN security approaches.
- **Towards P4-programmed switches:** We noticed from the literature reviewed that all researchers used default OpenFlow-enabled switches in their studies, except for [65], who employed programmable switches to work with a dynamic and flexible network. Utilizing P4-programmed switches within the SDN architecture could lower the controller’s overhead in case of DDoS attacks. Therefore, it is one of the promising future research directions for SDN network security defense approaches.

## 6. Conclusions and Limitations

This study provided an overview of the SDN architecture model, an example of the OpenFlow forwarding process, DDoS attacks on SDN networks, and a brief insight into the ML and DL techniques. In addition, this study formed six research questions related to ML, DL, and hybrid-based approaches (a combination of ML and DL) to detect and mitigate SDN DDoS attacks. The method used to answer those questions is the systematic literature review, providing a comprehensive analysis and synthesis of eight years of literature from 2014 to 2022, resulting in 70 studies being selected as primary studies relevant to the research questions. The studies that did not fulfill the inclusion and exclusion criteria or did not match the quality assessment levels were excluded.

Moreover, the significant findings of this SLR show that the number of publications is progressively increasing, particularly from 2020 onward, and we find that DDoS attacks have recently been around for a few years. In addition, we found that most of the literature studies used ML techniques (53%) for analysis, followed by DL (38%) and hybrid methods (9%). ML and DL-based detection techniques are commonly categorized as ensemble, hybrid, or single. However, ensemble-based ML and DL techniques are the most promising for detecting and mitigating SDN DDoS attacks. Another finding is that this SLR examined the network simulators and tools used to implement and design their approaches. We discovered that most researchers utilize the Mininet network emulator as an SDN testbed environment with a Python-based POX controller. Furthermore, the researchers mainly utilized Hping3 and Scapy hacking tools to generate DDoS attacks or normal traffic generation. Lastly, the researchers utilized the Wireshark network analyzer to collect the network traffic flows for further processing.

Furthermore, this SLR sheds light on the evaluation metrics researchers used to evaluate and validate their approaches. We found two main classes of performance evaluation metrics: first, the detection performance metrics contain all the measure evaluation metrics (i.e., confusion matrix, ROC, AUC, and detection accuracy, which are the most commonly used in the literature studies). Second, the computational performance metrics evaluate their approaches based on training and testing time, CPU utilization, and throughput. In addition, this SLR reveals that most of the literature studies prefer to generate their datasets, and there are a few publicly available realistic datasets. Finally, the SLR presented the extracted challenges, open-issue perspectives, research gaps, and feature directions to identify and address these aspects by the researchers for advancing research and development in detecting SDN DDoS attacks.

This SLR is limited to ML, DL, and hybrid-based approaches. However, other approaches based on information theory, blockchain, and other techniques, also need consideration in the future. This SLR raised many essential research questions, but not all techniques are covered or all questions answered. Instead, we tried our best to bridge the open research issues and perspectives, providing future directions and identifying the gaps in the literature studies as guidance for future research. In future works, we plan to extend this SLR to cover the newly published studies from 2022 onwards and conduct another SLR on SDN DDoS based on information theory and blockchain-based approaches.

## Figures and Tables

**Table 1:** Qualitative comparison of the previous SLRs.

Publication Year & Ref.	SLR	Approaches			Architecture SDN	OpenFlow Forwarding	DDoS Attack in SDN	Number of Online Databases	Time Span (Coverage Years)	Concentrated on Specific SDN Datasets
		ML	DL	Hybrid						
[8], 2020	✓	✓	✓	✗	✓	✓	✓	-	-	✗
[10], 2021	✓	✓	✗	✗	✓	✗	✓	4	2015–2021	✗
[11], 2021	✓	✓	✗	✗	✗	✗	✗	2	2019–2020	✗
[12], 2022	✓	✓	✓	✗	✓	✗	✓	8	Till April–2022	✗
[13], 2022	✓	✓	✓	✗	✓	✗	✓	5	2013–2020	✗
[14], 2023	✓	✓	✓	✓	✗	✗	✗	5	2018–2022	✗
This SLR, 2023	✓	✓	✓	✓	✓	✓	✓	8	2014–2022	✓

(✓): Addressed, (✗): Unaddressed, (-): Not clearly mentioned.

**Table 2:** Summary of the inclusion and exclusion criteria.

No.	This SLR Include Articles That Are:	This SLR Exclude Articles That Are:
1	From conferences, journals, and book chapters are written in the English language.	Written in other languages.
2	Listed at one of the database sources.	Not accessible full version.
3	Related to DDoS attacks detection and mitigation approach against SDN network.	Related to SDN DDoS attacks on IoT, cloud computing, 5G networks, mobile networks, wireless networks, and Ad hoc networks or DDoS attacks on conventional networks.
4	Empirical or experimental studies since the systematic reviews are generally concentrated on them.	Duplicated and unrelated (i.e., review/survey research papers, books, editorials, and not accessible) studies.
5	Published up to 2022.	Not within the search period.
6	Related to research questions.	Not related to research questions or score less than or equal to 3.5 in the quality assessment criteria.

**Table 3:** Online database sources.

No.	Online Database	Search Within	Links
1	IEEE Xplore.	Title, Abstract, Keywords	(https://ieeexplore.ieee.org, accessed on 1 January 2022).
2	Scopus-Elsevier.	Title, Abstract, Keywords	(https://www.scopus.com, accessed on 1 January 2022).
3	Taylor & Francis Online.	Title, Abstract, Keywords	(https://www.tandfonline.com, accessed on 1 January 2022).
4	Science Direct.	Title, Abstract, Keywords	(https://www.sciencedirect.com, accessed on 1 January 2022).
5	Web of Science (WoS).	Title, Abstract, Full Text	(https://www.webofknowledge.com, accessed on 1 January 2022).
6	Wiley Online Library.	Title, Abstract, Keywords	(https://onlinelibrary.wiley.com, accessed on 1 January 2022).
7	Springer Link.	Title, Abstract, Full Text	(https://link.springer.com, accessed on 1 January 2022).
8	ACM Digital Library.	Title, Abstract, Keywords	(https://dl.acm.org, accessed on 1 January 2022).

**Table 4:** Quality assessment (QA) criteria.

	Checklist Questions
Design	1. Are the objectives expressed clearly? 2. Is the topic addressed in the paper associated with this SLR? 3. Are the studies capable of answering any one of the research questions? 4. Are the study evaluation metrics fully defined?
Conduct	5. Is the dataset of the research paper clearly described? 6. Are the implementation methods sufficiently represented?
Analysis	7. Are the analysis of the results sufficiently explained? 8. Are the analysis of the results compared with existing approaches?
Conclusion	9. Are the research limitations stated?

**Table 5:** Extraction of data items of primary studies and their descriptions.

No.	Data Extracted	Description
1	Study ID	Specific identity numbers for each study.
2	Bibliographic Info	Title, author, publication source, and the publication year (up to 31 Mar 2022).
3	Type of publication	Conference or journal paper.
4	Study objective(s)	Aim of the study.
5	Method used.	The method used by the study (i.e., ML, DL, and hybrid (both ML and DL)).
6	Datasets type.	The dataset used by the study (i.e., benchmark dataset and realistic or unrealistic datasets).
7	Evaluation Metrics.	(i.e., detection performance/efficiency metrics or computational performance metrics).
8	Study finding.	Results of the study.
9	Study limitations.	Limitations of the study.
10	Study experiment.	Network simulators, hacking tools, and experimental platforms.

**Table 6:** Summarized ML-based approaches for detecting DDoS attacks in SDN Networks.

Ref.	ML-Based Approaches			Realistic Dataset	Feature Selection Technique *(f)*	Deployment of Detection Approach		DDoS Attack Techniques		Rates of DDoS Attacks		Detection Accuracy		Limitation(s)
	**Ẹ**	**Ḥ**	**Ṣ**			**In**	**Out**	**Ḍ**	**Ṃ**	**High**	**Low**	**High**	**Low**	
[59]	✓	✗	✗	✗	✓	✓	✗	✓	✓	✓	✗	✓	✗	The proposed approach was evaluated with unrealistic datasets, which did not reflect the characteristics of the SDN network.
[60]	✓	✗	✗	✗	✓	✓	✗	✓	✗	✓	✗	✓	✗	The proposed approach was evaluated with unrealistic datasets, which did not reflect the characteristics of the SDN network.
[61]	✓	✗	✗	✓	✓	✓	✗	✓	✗	✓	✗	✓	✗	Implementing the proposed model on a real SDN network is superior to testing its performance in detecting such attacks.
[62]	✓	✗	✗	✗	✓	✓	✗	✓	✗	✓	✗	✗	✓	The proposed approach was evaluated with unrealistic datasets, which did not reflect the characteristics of the SDN network. The ensemble approach achieved low performance.
[63]	✗	✓	✗	✓	✓	✓	✗	✓	✗	✓	✗	✓	✗	The approach is limited to high DDoS attacks, which are manageable to predict with high accuracy due to high forged traffic
[64]	✗	✓	✗	✗	✓	✓	✗	✓	✗	✓	✗	✓	✗	The approach was trained and tested with an unrealistic dataset, which does not reflect the character of the SDN network environment.
[65]	✗	✓	✗	✓	✓	✗	✓	✓	✗	✓	✗	✓	✗	The DAD is limited to SYN DDoS flood attacks on a data plan since the DDoS attacks against the SDN controller have a global impact. The proposed model was trained and tested using a small dataset.
[66]	✗	✓	✗	✗	✓	✓	✗	✓	✓	✓	✗	✓	✗	The proposed model was tested and trained with unrealistic datasets, which do not reflect the characteristics of SDN networks
[67]	✗	✓	✗	✓	✓	✓	✗	✓	✗	✓	✓	✓	✗	The proposed approach is limited to TCP-SYN flood attacks. The proposed method has been evaluated with a small dataset.
[68]	✗	✓	✗	✓	✓	✓	✗	✓	✗	✓	✗	✓	✗	It needs to be tested on a real SDN testbed, which would be preferable. It is limited to high-rate DDoS attacks, which are easy to detect due to massive network traffic flow.
[69]	✗	✓	✗	✓	✓	✓	✗	✓	✗	✓	✗	✓	✗	It was trained with the default settings because MLworks best when hyper-parameters or control parameters are tuned or optimized.
[70]	✗	✓	✗	✓	✓	✓	✗	✓	✓	✓	✗	✓	✗	The approach does not compare the results with other approaches. The proposed system runs on the controller, which adds unnecessary load and overhead to the controller.
[71]	✗	✓	✗	✗	✓	**-**	**-**	✓	✗	✓	✗	✓	✗	The remaining algorithms achieve low performance. The proposed method was evaluated using an unrealistic dataset, which does not reflect the characteristics of the SDN network environment.
[72]	✗	✓	✗	✗	✓	✓	✗	✓	✗	✓	✗	✗	✓	The method achieves deficient performance; for example, an accuracy rate of 78% and 85% for DT and SVM, respectively. The proposed method was evaluated using an unrealistic dataset, which does not reflect the characteristics of the SDN network environment.
[73]	✗	✓	✗	✓	✓	✓	✗	✓	✗	✓	✗	✓	✗	The remaining ML classifiers achieve relatively low performance regarding detection accuracy. There is no information about the proposed approach’s false positive rate.
[74]	✗	✓	✗	✗	✓	✗	✓	✓	✓	✗	✓	✗	✓	The proposed approach achieves a low detection accuracy of 95%. The proposed model was evaluated using an unrealistic dataset that does not reflect the characteristics of the SDN network environment.
[75]	✗	✓	✗	✓	✓	✓	✗	✓	✓	✓	✗	✓	✗	The framework is evaluated to detect high-rate DDoS attacks, which are easy to detect due to the massive amount of forged traffic. The proposed framework increases the controller’s workload.
[76]	✗	✓	✗	✗	✓	✓	✗	✓	✓	✓	✗	✓	✗	The proposed system runs on the controller, which adds unnecessary load and overhead to the controller. The proposed approach suffers from high processing and communication overhead.
[77]	✗	✓	✗	✓	✓	✓	✗	✓	✓	✓	✗	✓	✗	The proposed method implemented on the SDN controller adds an unnecessary burden in the case of DDoS attacks
[78]	✗	✓	✗	✓	✗	**-**	**-**	✓	✗	✓	✗	✗	✓	The proposed approach still achieves lower performance for detecting DDoS attacks, and needs to execute the approach on a real SDN network to test its performance in detecting such attacks.
[79]	✗	✓	✗	✗	✓	✓	✗	✓	✗	✓	✗	✗	✓	The proposed approach reveals low performance and needs improvement. The proposed approach was evaluated with an unrealistic dataset that does not reflect the characteristics of the SDN network.
[80]	✗	✓	✗	✓	✓	**-**	**-**	✓	✗	✓	✗	✓	✗	The proposed approach showed that the controller DDoS attack has the lowest classification results (for SVM and MLP, less than 90% accuracy rates) than flow-table and bandwidth attacks. There is no information about the proposed approach’s false positive rate.
[81]	✗	✓	✗	✗	✓	✗	✓	✓	✓	✓	✗	✓	✗	The defense system was evaluated, tested, and trained on an unrealistic dataset that does not reflect the characteristics of the SDN network environment.
[82]	✗	✓	✗	✓	✓	✓	✗	✓	✓	✓	✗	✓	✗	The proposed model runs at the controller, adding unnecessary load and overhead. The defense system was evaluated, tested, and trained on an unrealistic dataset that does not reflect the characteristics of the SDN network environment.
[83]	✗	✓	✗	✗	✓	✓	✗	✓	✗	✓	✗	✓	✗	The proposed approach implemented at the SDN controller increases the controller overhead in case of DDoS attacks.
[84]	✗	✗	✓	✓	✓	✓	✗	✓	✓	✓	✗	✓	✗	The proposed approach runs on the SDN controller as an application system, adding unnecessary load and overhead, particularly during DDoS attacks against the controller.
[85]	✗	✗	✓	✓	✓	**-**	**-**	✓	✗	✓	✗	✗	✓	The ASVM method achieves low performance for detecting DDoS attacks.
[86]	✗	✗	✓	✓	✓	✓	✗	✓	✗	✗	✓	✓	✓	The proposed approach achieves a low detection accuracy of 95.80%. The method’s detection performance is worse under different attack rates.
[87]	✗	✗	✓	✓	✓	✓	✗	✓	✗	✓	✗	✗	✓	The proposed approach implemented at the controller adds unnecessary load and overhead. It also needs to be improved in terms of detection accuracy.
[88]	✗	✗	✓	✗	✗	-	-	✓	✗	✓	✗	✓	✓	The performance of the proposed approach achieved low accuracy. Furthermore, the proposed approach was tested and trained using an unrealistic dataset that does not reflect the characteristics of the SDN network environment.
[89]	✗	✗	✓	✓	✓	✓	✗	✓	✗	✓	✗	✓	✗	The proposed approach on SDN switches makes the proposed method suffer from a scalability issue since the scheme must be implemented on every switch.
[90]	✗	✗	✓	✓	✓	✓	✗	✓	✓	✓	✗	✓	✗	The proposed model suffers from processing and communication overhead at the SDN controller. The proposed method was evaluated and trained on an unrealistic NSL-KDD dataset that does not reflect the characteristics of the SDN network environment.
[91]	✗	✗	✓	✓	✗	✓	✗	✓	✗	✓	✗	✓	✗	The proposed model runs at the controller, which adds excessive load and overhead of the SDN controller in the event of DDoS attacks. There is a lack of information about the tested and training datasets.
[92]	✗	✗	✓	✓	✓	✓	✗	✓	✓	✓	✗	✗	✓	The proposed approach shows a low detection accuracy of 96.13% because the proposed approach suffers from high false-positive and false-negative effects.
[93]	✗	✗	✓	✗	✓	✗	✗	✓	✗	✓	✗	✓	✗	The proposed approach has been evaluated with an unrealistic dataset, which does not reflect the characteristics of the SDN network environment. The proposed approach needs to be evaluated in terms of false-positive rates or any other evaluation metrics.
[94]	✗	✗	✓	✗	✓	✗	✗	✓	✗	✓	✗	✓	✗	The OpenFlow switches of the data layer have an attack-detection module that can analyze all packet flows that come through the switches, which takes more time to process and classify.
[95]	✗	✗	✓	✓	✓	✗	✓	✓	✓	✓	✗	✓	✗	This approach still suffers from overhead in the case of high DDoS attack flows because the SDN controller collects all flows from the switches for detection purposes, which results in congestion and degradation of the response time.

ML-based approaches: (Ẹ) ensemble, (Ḥ) hybrid, (Ṣ) single. Realistic dataset: (✓) realistic, (✗), unrealistic dataset. Deployment of detection approach: (In) inside or (Out) outside the SDN controller. DDoS attack techniques: (Ḍ), detection, (Ṃ), mitigation. Detection accuracy: High detection accuracy ≥ 98%, for low detection accuracy ≤ 97%. (✓): addressed, (✗): not addressed, and (-) not specified.

**Table 7:** Summary of DL-based approaches for detecting DDoS attacks in SDN networks.

Ref.	DL-Based Approaches			Realistic Dataset	Feature Selection Technique *(f)*	Deployment of Detection Approach		DDoS Attack Techniques		Rates of DDoS Attacks		Detection Accuracy		Limitation(s)
	**Ẹ**	**Ḥ**	**Ṣ**			**In**	**Out**	**Ḍ**	**Ṃ**	**High**	**Low**	**High**	**Low**	
[96]	✓	✗	✗	✗	✗	-	-	✓	✗	✓	✗	✓	✗	The proposed method was evaluated using an unrealistic dataset, which does not reflect the characteristics of the SDN network environment.
[97]	✓	✗	✗	✗	✓	-	-	✓	✗	✓	✗	✓	✗	The proposed approach was evaluated using an unrealistic dataset, which does not reflect the characteristics of the SDN network environment.
[98]	✓	✗	✗	✗	✓	-	-	✓	✗	✓	✗	✓	✗	The proposed ensemble model was evaluated, tested, and trained on an unrealistic dataset that does not reflect the characteristics of the SDN network environment.
[99]	✗	✓	✗	✓	✓	-	-	✓	✗	✓	✗	✓	✗	The hybrid approach achieved low accuracy but relatively high false-positive and false-negative rates.
[100]	✗	✓	✗	✗	✓	✓	✗	✓	✗	✓	✗	✓	✗	The approach is limited to detecting high-rate DDoS attacks. The proposed method was evaluated using an unrealistic dataset that does not reflect the characteristics of the SDN network environment.
[101]	✗	✓	✗	✓	✓	✓	✗	✓	✗	✗	✓	✓	✗	The dataset was not presented clearly and lacked information about low-rate DDoS attacks. Implementing the hybrid model in an SDN system is preferable to test its performance in detecting such attacks.
[102]	✗	✓	✗	✓	✓	✓	✗	✓	✗	✓	✗	✓	✗	The proposed approach performs better when the model detects the entire dataset of attacks. The proposed model runs at the controller, which adds unnecessary load and overhead to the controller.
[103]	✗	✓	✗	✓	✗	✓	✗	✓	✗	✓	✗	✗	✓	The proposed approach was evaluated using an unrealistic dataset and achieved low detection accuracy. The proposed model runs at the controller, which adds unnecessary load and overhead to the controller.
[104]	✗	✓	✗	✗	✓	✓	✗	✓	✗	✓	✗	✓	✗	The proposed approach was evaluated and trained using an unrealistic dataset, which does not reflect the characteristics of the SDN network environment. In addition, the proposed model runs at the controller, adding unnecessary load and overhead.
[105]	✗	✓	✗	✗	✓	✓	✓	✓	✗	✓	✗	✗	✓	The proposed system was evaluated with NSL-KDD dataset and achieved low detection accuracy.
[106]	✗	✓	✗	✗	✓	✓	✗	✓	✓	✓	✗	✓	✗	The proposed approach is trained, tested, and evaluated using an unrealistic dataset, which does not reflect the characteristics of the SDN network environment. The proposed approach achieved low detection accuracy of 89% for the NSL-KDD dataset. The proposed model runs at the controller, adding unnecessary load and overhead.
[107]	✗	✓	✗	✗	✓	✗	✓	✓	✗	✓	✗	✓	✗	The proposed approach was evaluated and trained using an unrealistic dataset, which does not reflect the characteristic of the SDN network environment.
[108]	✗	✓	✗	✗	✓	✓	✗	✓	✓	✓	✗	✗	✗	The detection rate of the proposed approach achieved a low detection accuracy. The proposed method was evaluated, tested, and trained using an unrealistic dataset that does not reflect the characteristics of the SDN network environment.
[109]	✗	✓	✗	✓	✓	✗	✓	✓	✗	✓	✗	✓	✗	The proposed method achieved low performance, particularly in the two-level method and information entropy, because it does not consider adaptive parameters
[110]	✗	✓	✗	✗	✓	-	-	✓	✗	✓	✗	✗	✓	The proposed method was evaluated using an unrealistic Dataset, which does not reflect the characteristics of the SDN network environment.
[111]	✗	✗	✓	✓	✓	✓	✗	✓	✗	✓	✗	✓	✗	The proposed system is complicated since it must first convert the selected features into RGB images to rescale the data and then forward them to the CNN classifier model, and the proposed system runs at the SDN controller, adding unnecessary overhead during DDoS attacks.
[112]	✗	✗	✓	✗	✓	✓	✗	✓	✓	✓	✗	✓	✗	The proposed system was evaluated using unrealistic datasets, which do not reflect the characteristics of the SDN network environment. The proposed method suffers from computational overhead and needs to improve the mitigation results.
[113]	✗	✗	✓	✓	✓	✓	✗	✓	✓	✓	✗	✓	✗	The proposed approach runs on the SDN controller, which adds unnecessary load and overhead, particularly during DDoS attacks against the controller.
[114]	✗	✗	✓	✓	✓	**-**	**-**	✓	✓	✓	✗	✓	✗	The proposed system needs to be tested in an SDN environment. The approach needs to be compared with the findings of other existing approaches.
[115]	✗	✗	✓	✓	✓	-	-	✓	✗	✓	✗	✗	✓	The proposed approach achieved a low detection accuracy.
[116]	✗	✗	✓	✗	✓	-	-	✓	✗	✓	✗	✓	✗	The proposed method was evaluated using an unrealistic dataset, which does not reflect the characteristics of the SDN network environment.
[117]	✗	✗	✓	✓	✓	✓	✗	✓	✗	✓	✗	✓	✗	The proposed approach must be evaluated using other evaluation metrics (i.e., false-positive rates) and compared with other existing approaches.
[118]	✗	✗	✓	✓	✓	✗	✓	✓	✓	✓	✗	✗	✓	Around 85% of the regular traffic reaches the server when the mitigation agent starts against the flooding attacks. The mitigation agent may not perform as a stand-alone IDS for detecting such attacks.
[119]	✗	✗	✓	✗	✓	-	-	✓	✗	✓	✗	✓	✗	The proposed system was tested and trained with an unrealistic dataset, which does not reflect the characteristics of the SDN network.
[120]	✗	✗	✓	✓	✓	✓	✗	✓	✗	✓	✗	✗	✓	The proposed DL-based DDoS detection model has low detection accuracy. The approach runs on the SDN controller, which adds unnecessary load and overhead, particularly when detecting DDoS attacks against the controller.
[121]	✗	✗	✓	✗	✓	✗	✓	✓	✗	✓	✗	✓	✗	The proposed approach achieved a low detection accuracy. The proposed method was evaluated and trained using an unrealistic dataset that does not reflect the characteristics of the SDN network environment.
[122]	✗	✗	✓	✓	✓	✓	✗	✓	✗	✓	✗	✗	✓	The proposed system’s performance needs to be improved.

DL-based approaches: (Ẹ) ensemble, (Ḥ) hybrid, (Ṣ) single. Realistic dataset: (✓) realistic, (✗), unrealistic dataset. Deployment of detection approach: (In) inside or (Out) outside the SDN controller. DDoS attack techniques: (Ḍ), detection, (Ṃ), mitigation. Detection accuracy: high detection accuracy ≥ 98%, for low detection accuracy ≤ 97%. (✓): addressed, (✗): not addressed, and (-) not specified.

**Table 8:** Summary of hybrid-based approaches for detecting DDoS attacks in SDN networks.

Ref.	Hybrid- Based Approaches	Realistic Dataset	Feature Selection Technique *(f)*	Deployment of Detection Approach		DDoS Attack Techniques		Rates of DDoS Attacks		Detection Accuracy		Limitation(s)
				**In**	**Out**	**Ḍ**	**Ṃ**	**High**	**Low**	**High**	**Low**	
[123]	✓	✓	✓	-	-	✓	✗	✓	✗	✓	✗	It needs to be implemented in an SDN environment to test its performance in detecting such attacks.
[124]	✓	✓	✓	✓	✗	✓	✗	✓	✗	✓	✗	The proposed approach runs at the controller, which adds unnecessary load and overhead to the controller and achieves low performance for application-layer DDoS attack.
[125]	✓	✗	✓	-	-	✓	✗	✓	✗	✓	✗	The proposed approach runs on the SDN controller, adding unnecessary load and overhead. The approach was trained and evaluated using an unrealistic dataset, which does not reflect the characteristics of the SDN network environment.
[126]	✓	✓	✗	✓	✗	✓	✓	✓	✗	✓	✗	The proposed anomaly detection and mitigation approach achieved high performance for the first scenario, and satisfactory performance for the second scenario. The proposed approach runs on the SDN application plane, which adds unnecessary load and overhead to the controller in the event of DDoS attacks against the SDN controller.
[127]	✓	✓	✓	-	-	✓	✗	✓	✗	✗	✓	The proposed approach achieved a relatively low detection accuracy of 90.5%. Implementing the proposed model in a real SDN network is superior to testing its performance in detecting such attacks and extending the binary classification into multi-class classification problems to identify network attacks.
[128]	✓	✗	✗	✓	✗	✓	✗	✓	✗	✗	✓	The proposed approach achieved low detection accuracy of 74.3% and 92.3% for SVM and DNN, respectively. The proposed method was evaluated, tested, and trained on an unrealistic dataset that does not reflect the characteristics of the SDN network environment.

Hybrid-based approaches: a combination ofML and DL-based approaches. Realistic dataset: (✓) realistic, (✗), unrealistic dataset. Deployment of detection approach: (In) inside or (Out) outside the SDN controller. DDoS attacks techniques: (Ḍ), detection, (Ṃ), mitigation. Detection accuracy: high detection accuracy ≥ 98%, for low detection accuracy ≤ 97%. (✓): addressed, (✗): not addressed, and (-) not specified.

## Data Availability

Not applicable.

## Appendix A. Quality Assessment (QA) Criterion

See Table A1.

**Table A1:** Quality Assessment (QA) Scores of Primary Studies.

Ref.	Study ID	Design				Conduct		Analysis		Conclusion	Total Score	QA Studies Level		
		Q1	Q2	Q3	Q4	Q5	Q6	Q7	Q8	Q9		High	Medium	Low
[59]	S1	1	1	1	1	1	1	1	1	1.5	8.5	✓	✗	✗
[60]	S2	1	1	1	1	0.5	0.5	0.5	0.5	0	6	✓	✗	✗
[61]	S3	0.5	1	1	1	0.5	0.5	0.5	0.5	0	5.5	✗	✓	✗
[62]	S4	0.5	1	1	0.5	0.5	0.5	0.5	0.5	0	5	✗	✓	✗
[63]	S5	1	1	1	1	0.5	1	1	1	0.5	8	✓	✗	✗
[64]	S6	1	1	1	0.5	0.5	0.5	0.5	0.5	0	5.5	✗	✓	✗
[65]	S7	1	1	1	1	0.5	0.5	0.5	0.5	0.5	6.5	✓	✗	✗
[66]	S8	1	1	1	1	1	0.5	0.5	0.5	0.5	7	✓	✗	✗
[67]	S9	1	1	1	1	0.5	0.5	0.5	0.5	0.5	6.5	✓	✗	✗
[68]	S10	1	1	1	1	1	0.5	0.5	0.5	0.5	7	✓	✗	✗
[69]	S11	1	1	1	1	0.5	1	1	0.5	0.5	7.5	✓	✗	✗
[70]	S12	1	1	1	1	1	0.5	1	0.5	0.5	7.5	✓	✗	✗
[71]	S13	1	1	1	1	0.5	0.5	0.5	0	0	5.5	✗	✓	✗
[72]	S14	0.5	1	1	0.5	0.5	0.5	0.5	0	0	4.5	✗	✗	✓
[73]	S15	1	1	1	1	0.5	0.5	0.5	0.5	0.5	6.5	✓	✗	✗
[74]	S16	1	1	1	1	0.5	0.5	1	0.5	0.5	7	✓	✗	✗
[75]	S17	1	1	1	1	0.5	0.5	0.5	0.5	0.5	6.5	✓	✗	✗
[76]	S18	1	1	1	1	0.5	0.5	1	0.5	0.5	7	✓	✗	✗
[77]	S19	1	1	1	1	0.5	1	1	0.5	0.5	7.5	✓	✗	✗
[78]	S20	0.5	1	1	0.5	0.5	0.5	0.5	0	0	4.5	✗	✗	✓
[79]	S21	1	1	1	1	1	1	1	0.5	0.5	8	✓	✗	✗
[80]	S22	1	1	1	1	0.5	0.5	0.5	0.5	0.5	6.5	✓	✗	✗
[81]	S23	0.5	1	1	1	0.5	0.5	0.5	0.5	0	5.5	✗	✓	✗
[82]	S24	1	1	1	1	1	1	1	0.5	0.5	8	✓	✗	✗
[83]	S25	0.5	1	1	0.5	0.5	0.5	0.5	0.5	0	5	✗	✓	✗
[84]	S26	0.5	0.5	1	0.5	0.5	0.5	0.5	0.5	0	4.5	✗	✗	✓
[85]	S27	1	1	1	1	0.5	0.5	0.5	0.5	0	6	✓	✗	✗
[86]	S28	1	1	1	1	0.5	0.5	0.5	0.5	1	7	✓	✗	✗
[87]	S29	1	1	1	1	1	0.5	0.5	0.5	0.5	7	✓	✗	✗
[88]	S30	0.5	1	1	0	0.5	0.5	0.5	0	0	4	✗	✗	✓
[89]	S31	1	1	1	1	0.5	1	1	0.5	0.5	7.5	✓	✗	✗
[90]	S32	1	1	1	1	0.5	0.5	1	1	0.5	7.5	✓	✗	✗
[91]	S33	1	1	1	1	0.5	1	1	0.5	0	7	✓	✗	✗
[92]	S34	1	1	1	1	0.5	0.5	0.5	0.5	0.5	6.5	✓	✗	✗
[93]	S35	1	1	1	1	0.5	0.5	0.5	0	0	5.5	✗	✓	✗
[94]	S36	1	1	1	1	0.5	1	1	0.5	1	8	✓	✗	✗
[95]	S37	1	1	1	1	0.5	1	1	0.5	1	8	✓	✗	✗
[96]	S38	1	1	1	1	1	0.5	0.5	0.5	0.5	7	✓	✗	✗
[97]	S39	1	1	1	0.5	0.5	0.5	0.5	0.5	0	5.5	✗	✓	✗
[98]	S40	0.5	1	1	0.5	0.5	1	1	0	0	4.5	✗	✗	✓
[99]	S41	1	1	1	1	0.5	0.5	0.5	0.5	0.5	6.5	✓	✗	✗
[100]	S42	1	1	1	1	0.5	0.5	1	1	0.5	7.5	✓	✗	✗
[101]	S43	0.5	1	1	1	0.5	0.5	0.5	0.5	0	5.5	✗	✓	✗
[102]	S44	1	1	1	1	0.5	0.5	0.5	0	0	5.5	✗	✓	✗
[103]	S45	1	1	1	1	0.5	0.5	0.5	0.5	0.5	6.5	✓	✗	✗
[104]	S46	1	1	1	0.5	0.5	0.5	0.5	0.5	0.5	6	✓	✗	✗
[105]	S47	1	1	1	1	0.5	1	0.5	0.5	0.5	7	✓	✗	✗
[106]	S48	1	1	1	1	0.5	0.5	0.5	1	0	6.5	✓	✗	✗
[107]	S49	1	1	1	1	0.5	0.5	0.5	0.5	0.5	6.5	✓	✗	✗
[108]	S50	1	1	1	1	0.5	0.5	0.5	0.5	0.5	6.5	✓	✗	✗
[109]	S51	1	1	1	1	1	1	1	0.5	0.5	8	✓	✗	✗
[110]	S52	1	1	1	1	0.5	0.5	0.5	0.5	0.5	6.5	✓	✗	✗
[111]	S53	1	1	1	1	1	1	1	0.5	0.5	8	✓	✗	✗
[112]	S54	1	1	1	1	1	1	0.5	0.5	0.5	7.5	✓	✗	✗
[113]	S55	1	1	1	1	1	1	1	0.5	0.5	8	✓	✗	✗
[114]	S56	0.5	1	1	0.5	0.5	0.5	0.5	0	0	4.5	✗	✗	✓
[115]	S57	1	1	1	1	0.5	0.5	0.5	0.5	0.5	6.5	✓	✗	✗
[116]	S58	1	1	1	1	0.5	0.5	0.5	0.5	0	6	✓	✗	✗
[117]	S59	0.5	1	1	0.5	0.5	0.5	0.5	0	0	4.5	✗	✗	✓
[118]	S60	1	1	1	1	0.5	0.5	0.5	0	0	5.5	✗	✓	✗
[119]	S61	1	1	1	1	0.5	0.5	0.5	0.5	0	6	✓	✗	✗
[120]	S62	1	1	1	1	0.5	0.5	0.5	0.5	0.5	6.5	✓	✗	✗
[121]	S63	1	1	1	0.5	0.5	0.5	0.5	0.5	0	5.5	✗	✓	✗
[122]	S64	0.5	1	1	0.5	0.5	0.5	0.5	0	0	4.5	✗	✗	✓
[123]	S65	1	1	1	1	1	1	1	1	1	9	✓	✗	✗
[124]	S66	1	1	1	1	0.5	1	1	0.5	0.5	7.5	✓	✗	✗
[125]	S67	1	1	1	0.5	0.5	0.5	0.5	0	0.5	5.5	✗	✓	✗
[126]	S68	1	1	1	1	0.5	0.5	0.5	0.5	0.5	6.5	✓	✗	✗
[127]	S69	1	1	1	1	1	0.5	0.5	0.5	0.5	7	✓	✗	✗
[128]	S70	1	1	1	1	0.5	0.5	0.5	0	0	5.5	✗	✓	✗
